# Carbon Oxidation State in Microbial Polar Lipids Suggests Adaptation to Hot Spring Temperature and Redox Gradients

**DOI:** 10.3389/fmicb.2020.00229

**Published:** 2020-02-20

**Authors:** Grayson M. Boyer, Florence Schubotz, Roger E. Summons, Jade Woods, Everett L. Shock

**Affiliations:** ^1^School of Earth and Space Exploration, Arizona State University, Tempe, AZ, United States; ^2^MARUM and Department of Geosciences, University of Bremen, Bremen, Germany; ^3^Department of Earth, Atmospheric and Planetary Science, Massachusetts Institute of Technology, Cambridge, MA, United States; ^4^Department of Chemistry, University of Nebraska-Lincoln, Lincoln, NE, United States; ^5^School of Molecular Sciences, Arizona State University, Tempe, AZ, United States

**Keywords:** geobiochemistry, intact polar lipid, redox gradient, hydrothermal system, microbial community, carbon oxidation state

## Abstract

The influence of oxidation-reduction (redox) potential on the expression of biomolecules is a topic of ongoing exploration in geobiology. In this study, we investigate the novel possibility that structures and compositions of lipids produced by microbial communities are sensitive to environmental redox conditions. We extracted lipids from microbial biomass collected along the thermal and redox gradients of four alkaline hot springs in Yellowstone National Park (YNP) and investigated patterns in the average oxidation state of carbon (Z_C_), a metric calculated from the chemical formulae of lipid structures. Carbon in intact polar lipids (IPLs) and their alkyl chains becomes more oxidized (higher Z_C_) with increasing distance from each of the four hot spring sources. This coincides with decreased water temperature and increased concentrations of oxidized inorganic solutes, such as dissolved oxygen, sulfate, and nitrate. Carbon in IPLs is most reduced (lowest Z_C_) in the hot, reduced conditions upstream, with abundance-weighted Z_C_ values between −1.68 and −1.56. These values increase gradually downstream to around −1.36 to −1.33 in microbial communities living between 29.0 and 38.1°C. This near-linear increase in Z_C_ can be attributed to a shift from ether-linked to ester-linked alkyl chains, a decrease in average aliphatic carbons per chain (nC), an increase in average degree of unsaturation per chain (nUnsat), and increased cyclization in tetraether lipids. The Z_C_ of lipid headgroups and backbones did not change significantly downstream. Expression of lipids with relatively reduced carbon under reduced conditions and oxidized lipids under oxidized conditions may indicate microbial adaptation across environmental gradients in temperature and electron donor/acceptor supply.

## 1. Introduction

There is ongoing interest in how geochemistry influences lipid compositions expressed in living communities of microorganisms. Lipids can provide valuable information about the environmental conditions experienced by the microbes that produced them (Summons and Walter, 1990; Pearson and Ingalls, 2013; Schouten et al., 2013). Structural diversity, longevity, and potential traceability have led to the extensive use of lipids in biogeoscience as biomarkers (Summons et al., 1999; Brocks and Pearson, 2005; Schouten et al., 2007a). However, interpreting lipid biomarkers can be challenging because they are often not specific to any single type of organism or set of geochemical conditions (Rashby et al., 2007; Pitcher et al., 2009; French et al., 2015). For this reason, it is useful to investigate how bulk lipid compositions change across a variety of natural systems and then look for patterns that are universally applicable.

Hot spring outflow channels provide particularly accessible locations for studying changes in lipid composition across strong temperature and chemical gradients. For instance, microbial communities in the submerged sediments and biofilms of boiling springs tend to express lipid compositions substantially different from those in a hot spring photosynthetic microbial mat downstream (Zeng et al., 1992; Schubotz et al., 2013). It is conceivable that temperature is not the only environmental stress governing distributions of lipid structural adaptations along a hot spring outflow channel. As is often the case in hydrothermal systems, temperature gradients coincide with gradients in water chemistry, such as pH, salinity, solute concentrations, and redox potential. Changes in lipid structures and compositions along these gradients likely reflect adaptation to the collective set of environmental conditions experienced by the microorganisms present.

Our goal was to identify patterns in the properties of microbial lipids that might be influenced by redox geochemistry. We decided to explore the average oxidation state of carbon (Z_C_) in lipids. In general, lower values of Z_C_ in a molecule represent more reduced carbon (e.g., −4 in CH_4_) while higher values indicate more oxidized carbon (e.g., +4 in CO_2_). Z_C_ was chosen as a metric primarily because thermodynamic or kinetic relationships with redox potential have been implicated in a variety of natural processes. Examples include the predicted energetic favorability of amino acids biosynthesis in submarine hydrothermal vents (Amend and Shock, 1998), preservation and degradation of organic matter in sediments and soils (Likens, 2010; LaRowe and Van Cappellen, 2011; Boye et al., 2017), oxidation rates of atmospheric organic aerosols (Kroll et al., 2011, 2015), and evolutionary convergence on proteomes inferred from metagenomes of microbial communities in natural systems (Dick and Shock, 2011, 2013; Dick, 2014; Dick et al., 2019; Fones et al., 2019), in aerobic and anaerobic nitrogen-fixing bacteria and archaea (Poudel et al., 2018), and in human cancer tissue (Dick, 2016, 2017). Further, several of these studies have implicated Z_C_ as a useful proxy for biosynthetic costs predicted from redox geochemistry (Amend and Shock, 1998; Dick and Shock, 2013; Dick et al., 2019). Thermodynamic properties do not exist for the full suite of lipid structures found along a hot spring outflow channel, so a similar assessment is not yet possible. However, we reasoned that if patterns in Z_C_ were evident, this would provide impetus for an eventual thermodynamic analysis to quantify lipid energy costs along thermal and chemical gradients.

We calculated Z_C_ values for intact polar lipids (IPLs) from eighteen sediment and microbial biomass samples collected from the outflow channels of four alkaline YNP hot springs: Bison Pool, Mound Spring, Empress Pool, and Octopus Spring. Further, we calculated the Z_C_ of lipid headgroups, backbones, and alkyl chains to better understand how these components influence changes in the Z_C_ of IPLs. These Z_C_ values could then be correlated with the temperature, chemical composition, and redox state of the surrounding environment. We hypothesized that lipids sampled closest to the hot, reduced source of each spring would have the most reduced carbon on average, while the cool, oxidized conditions downstream would be characterized by lipids with more oxidized carbon. This general pattern aligns with observations of amino acid compositions inferred from metagenomes along the outflow channel of Bison Pool (Dick and Shock, 2011, 2013), and the strong correlations found between the Z_C_ of metagenomes and metatranscriptomes across natural redox gradients in a variety of microbial mats, terrestrial and marine hydrothermal systems, and hypersaline lakes (Dick et al., 2019). If compositions of microbial lipids display similar trends across a variety of natural systems, patterns in Z_C_ preserved in lipid biomarkers could potentially present a tantalizing target for inferring paleoredox.

## 2. Methods

### 2.1. Water Chemistry

Temperature, conductivity, and pH were measured in the field as close to sampling locations as possible and before sample collection. Temperature and conductivity were measured with a YSI model 30 meter. Sample pH was measured with a WTW brand 3300i or 3110 model pH meter with WTW probe calibrated daily with pH 4, 7, and 10 buffer solutions at ambient temperature. Concentrations of dissolved oxygen and total sulfide were obtained from unfiltered water samples in the field using a Hach 2400 or 2800 portable spectrophotometer with Hach reagents and protocols. Water samples collected for laboratory analyses were filtered in the field with Supor^TM^ (Pall Corporation) 0.2 μm polyethersulfone (PES) syringe filters into 30 mL HDPE Nalgene bottles and stored at −20°C. Concentrations of total ammonium, nitrate, nitrite, and sulfate were obtained by ion chromatography on two Dionex DX-600 systems; one for the analysis of cations and the other for anions. Suppressor columns on both systems were regenerated with deionized water to improve the signal-to-noise ratio. The anion analysis system was equipped with a potassium hydroxide eluent generator, carbonate removal device, and AS11-HC/AG11-HC columns. Columns were equilibrated with 5 mM hydroxide for 10 min before each injection. The injection volume was 100 μL for anions. Using a constant flow rate of 1.0 mL/min, the eluent hydroxide concentration was held isocratically at 5 mM for 5 min, then increased over the course of 31 min with a non-linear gradient (Chromeleon curve 8). The cation analysis system was equipped with CS-16 and CG-16 columns. Cation samples were acidified with 6 N methanesulfonic acid (MSA) to 19 mM final concentration. The injection volume was 75 μL for cation analysis. The columns were eluted isocratically with 19 mM MSA and a flow rate of 0.5 mL/min. Ion concentrations were obtained by comparison to calibration curves created using mixed ion standards (Environmental Express, Charleston, SC, USA). Quantification accuracy was verified by the inclusion of mixed ion-quality control standards (Thermo Scientific, Waltham, MA, USA) before, between, and after samples in each tray.

### 2.2. Sample Collection and Preparation

Samples for lipid analysis were collected with ethanol-cleaned spatulas or forceps into sterile 15 mL falcon tubes. Sediment and microbial mat samples were collected to ~1 cm depth, while samples BP1, BP2, and OS1 were taken from streamer biofilm communities clinging to gravel below the surface of the outflow water. Samples were frozen on dry ice in the field before storage in a −80°C freezer. Frozen samples were freeze-dried and homogenized with a sterile mortar and pestle. Lipid extractions were carried out using a modified version of the Bligh and Dyer procedure (White and Ringelberg, 1998). Briefly, 0.5–2 g sediment or 200–800 mg biofilm was dissolved in a mixture of methanol (MeOH), dichloromethane (DCM), and 50 mM phosphate buffer at pH 7.4 (2:1:0.8 v/v). The mixture was sonicated for 10 min and then centrifuged for 10 min at 2,000 rpm. The supernatant was collected and the remaining sediment or biofilm underwent one more extraction with the same solvent proportions, followed by two more times with a mixture of MeOH, DCM, and 50 mM trichloroacetic acid buffer at pH 2 (2:1:0.8) to aid extraction of glycerol dialkyl glycerol tetraether (GDGT) lipids (Nishihara and Koga, 1987), and one more time with a mixture of 3:1 DCM:MeOH to account for less polar lipids. A liquid-liquid extraction was performed by adding equal volumes of water and DCM to the pooled supernatant, which was then mixed and allowed to separate into aqueous polar and organic non-polar phases. The non-polar organic phase was collected and the remaining aqueous phase was washed with equal parts DCM for two additional rounds of liquid-liquid extraction. The resulting total lipid extract (TLE) was dried under N_2_ and redissolved in 9:1 DCM and MeOH for later analyses.

### 2.3. HPLC-MS

Aliquots of the TLE were chromatographically separated on an Agilent 1200 series high-performance liquid chromatograph (HPLC) equipped with a Waters Acquity Ultra Performance Liquid Chromatography ethylene bridge hybrid (BEH) amide column according to the hydrophilic interaction chromatography (HILIC) method described in Wörmer et al. (2013). Mobile phases included solvent A, a mixture of acetonitrile, DCM, formic acid, and ammonia (750:250:0.015:0.15 v/v) and solvent B, a mixture of MeOH, H_2_O, formic acid, and ammonia (500:500:4:4). The initial eluent was 99% solvent A and 1% solvent B that was brought to 5% B with a linear gradient over 4 min. The gradient continued to 25% B over 18.5 min, then to 40% over 0.5 min and held isocratically for 3.5 min. The flow rate was held constant at 0.4 mL min^−1^ throughout each run. Mass spectral analysis of IPLs was performed in positive ion mode on an Agilent 6520 Accurate-Mass Quadrupole Time-of-Flight (Q-TOF) mass spectrometer equipped with an electrospray ionization source.

### 2.4. Interpretation of Mass Spectra

IPLs were identified by the exact mass (M) of their parent ion, i.e., the intact lipid molecule with either a proton adduct [M + H]^+^ or ammonium ion adduct [M + NH_4_]^+^, and by comparing mass-to-charge (m/z) fragmentation patterns to previously published data as described in Sturt et al. (2004). Table 1 summarizes references used for structural elucidation or mass spectral interpretation of IPLs. Structures exceeding the analytical window (m/z >2000) were not detected, potentially leading to the exclusion of some higher molecular weight lipids.

**Table 1:** Observed polar lipids, headgroup formulae and their Z_C_ values, references used for identification, and assigned HPLC-MS quantification standards.

**Headgroup**			**Backbone-chain** linkage types^*^	**Ref^‡^**	**RF^**§**^**	**Headgroup**			**Backbone-chain** linkage types^*^	**Ref^‡^**	**RF^**§**^**
Abbreviation^*^	**Formula^†^**	**Z_**C**_**				Abbreviation^*^	**Formula^†^**	**Z_**C**_**			
**Glycolipids**						**Phospholipids**					
1G	C_6_H_11_O_5_	$-0.16\bar{6}$	DEG, AEG, DAG	a, b	1	APT	C_5_H_13_NO_6_P	−0.600	DEG, AEG, DAG	a, b	3
			GDGT	b, c	2	DPG	C_3_H_8_O_7_P_2_	$-1.33\bar{3}$	DAG	a	7
			CER	a, d	3	PC	C_5_H_14_NO_3_P^+^	−1.800	DAG	a, b	3
2G	C_12_H_21_O_10_	$-0.08\bar{3}$	DEG, AEG, DAG	a, b	4	PDME	C_4_H_11_NO_3_P	−1.750	DAG	j	8
			GDGT	b, c	2	PE	C_2_H_7_NO_3_P	−1.500	DAG, DEG, CER	a, c, d	9
3G	C_18_H_31_O_15_	$-0.05\bar{5}$	DEG, DAG	a	4	PG	C_3_H_8_O_5_P	−1.000	DAG	a, b	10
			GDGT	b, c	2	PME	C_3_H_9_NO_3_P	$-1.66\bar{6}$	DAG	j	11
2G-NAcG-G	C_24_H_41_NO_19_	0.000	DAG, DEG	b, e	3	PS	C_3_H_7_NO_5_P	0.333	DAG	a	12
3G-NAcG-G	C_30_H_51_NO_24_	0.000	DEG	f	3						
4G	C_24_H_41_O_20_	−0.042	GDGT	b, c	2	**Aminolipids**					
GA	C_6_H_9_O_6_	0.500	DAG	g	1	BL	C_7_H_15_NO_2_^+^	−1.000	DAG	b, k	13
G-GA	C_12_H_19_O_11_	0.250	DAG	f	4	OL	C_5_H_10_NO_2_	−0.600	FA-OH-FAm(-OH)	l, b, g	13
G-NG	C_12_H_22_NO_9_	$-0.08\bar{3}$	DAG, DEG	h	3	TM-KL	C_9_H_19_NO_2_^+^	$-1.22\bar{2}$	FA-OH-FAm	f	13
NG-GA	C_12_H_20_NO_10_	0.250	DAG, AEG, DEG	f	3	TM-OL	C_8_H_17_NO_2_^+^	−1.125	FA-OH-FAm(-OH)	m	13
SQ	C_6_H_11_O_7_S	$-0.16\bar{6}$	DAG	b	5						
						**Unidentified**					
**Glycophospholipids**						“223”	C_7_H_12_NO_6_	0.429	DAG	f	3
1G-P	C_6_H_12_O_8_P	$-0.16\bar{6}$	GDGT	b, c	2						
2G-P	C_12_H_22_O_13_P	$-0.08\bar{3}$	DEG	a, c	6	**Other**					
			GDGT	b, c	2	hydroxyl “H”	H	Special^∥^	GDGT	b, c	2
3G-P	C_18_H_32_O_18_P	$-0.05\bar{5}$	GDGT	b, c	2						
G-MeNG-G-P	C_19_H_35_NO_17_P	−0.158	DEG	f	3						
G-NG-G-P	C_18_H_33_NO_17_P	$-0.05\bar{5}$	DEG	f	3						
MeNG-G-P	C_13_H_25_NO_12_P	−0.231	DEG	f	3						
NAcG-P	C_12_H_21_N_2_O_10_P	0.000	DAG, DEG	b, i	3						
NG-G-P	C_12_H_23_NO_12_P	$-0.08\bar{3}$	DEG	f	3						
PI	C_6_H_12_O_8_P	$-0.16\bar{6}$	DAG, AEG, DEG	a, b	6						
			CER	a, b, d	3						

* See text for abbreviations.

**Figure 1:**
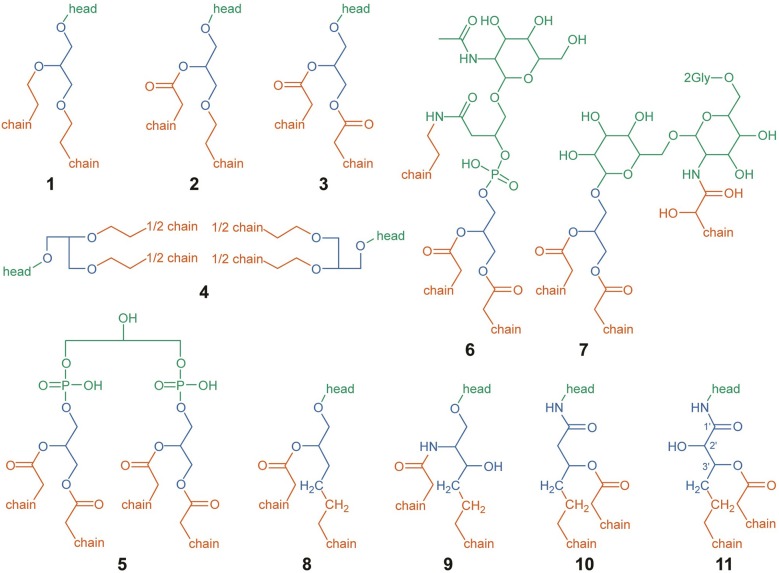
Structural designations used for IPL headgroups (green), backbones (blue), and alkyl chains (orange), for the sake of calculating abundance-weighted average properties and chemical formulae. Structures are depicted for DEG **(1)**, AEG **(2)**, DAG **(3)**, GDGT **(4)**, DPG **(5)**, NAcG-P-DAG **(6)**, 2GNAcG-G-DAG **(7)**, 1,2-alkanediol **(8)**, CER **(9)**, FA-OH-FAm **(10)**, and FA-OH-FAm-OH **(11)**. Abbreviations are defined in the text. For structures **(1–4)** and **(8–11)**, the chemical structure of the headgroup is represented by “head.” Putative headgroup structures are shown for **(6)** and **(7)**. Only the chemical structure of the first two carbons of each alkyl chain are shown; with “chain” representing the rest. In FA-OH-FAm-OH **(11)**, backbone-alkyl chain esterification may occur on either the 2′ or 3′ hydroxyl group (Diercks et al., 2015).

† Formulae correspond to elemental abundances contained in headgroups according to the division scheme depicted in Figure 1 and described in the methods.

‡ References used for structural elucidation and/or mass spectral interpretation; a. Sturt et al. (2004); b. Schubotz et al. (2013); c. Yoshinaga et al. (2011); d. Karlsson et al. (1998); e. Ferreira et al. (1999); f. this work (see Supplementary Material for mass spectral interpretation); g. Diercks et al. (2015); h. Schubotz et al. (2015); i. Yang et al. (2006); j. Wang et al. (2015); k. Benning et al. (1995); l. Zhang et al. (2009); m. Moore et al. (2013).

§ IPL standard used to determine analytical response factors. Numbers correspond to commercially-available standards reported in Table S1.

∥ *This headgroup does not contain carbon and therefore does not have a Z_C_ value, though it still contributes to the Z_C_ calculated for all IPLs or headgroups in a sample*.

While headgroup moiety identities, number of chains, backbone-chain linkage types, unsaturations, and aliphatic chain carbons were inferred based on mass spectra, other structural information was not obtained, such as positions of double bonds in alkyl chains or glycosidic bonds linking sugar headgroup moieties. Branching in non-isoprenoidal chains, such as those found in iso- and anteiso fatty acids, could not be determined from their straight-chain counterparts, necessitating “number of aliphatic carbons per chain” as a metric of alkyl chain carbon content rather than “chain length,” which implies distance spanned by straight or branching chains. Chain cyclizations in non-GDGT IPLs, such as cyclopropane fatty acids synthesized by certain bacteria (Grogan and Cronan, 1997), could not be discerned from unsaturations, as both types of chain modification have two fewer hydrogen atoms relative to a saturated straight chain. As such, these were counted as unsaturations. It is important to note that knowledge of the carbon positions of alkyl chain modifications, or whether a 2 Da loss is due to unsaturation or cyclization, and other fine details of molecular configuration are not required for the calculation of lipid Z_C_. This is because Z_C_ depends solely on elemental abundances, oxidation states of non-carbon elements, and molecular charge. Additional discussion regarding IPLs potentially underrepresented in this study can be found in the Supplementary Material.

### 2.5. Lipid Quantification

IPLs were quantified based on manual peak integration of identified parent ions. The mole fraction of the *i*th IPL in a sample, *x*_*i*_, was calculated using

(1) $$\begin{array}{l} x_{i}=\frac{I_{i}\cdot RF_{i}^{-1}\cdot \;mi_{i}^{-1}}{\sum_{i}\left(I_{i}\cdot RF_{i}^{-1}\cdot \;mi_{i}^{-1}\right)}, \end{array}$$

where *I*_*i*_ stands for the manually integrated MS peak area, *RF*_*i*_ indicates the assigned analytical response factor, and *mi*_*i*_ designates the monoisotopic mass, all taken for the *i*th IPL parent ion.

Analytical response factors were applied in this study to partially account for differences in IPL ionization efficiency. Response factors were estimated by taking the linear slope of the injected masses vs. peak intensity for a small suite of co-analyzed commercially-available IPL standards. Because authentic standards are not available for every observed IPL structure, response factors were assigned based primarily on the similarity of headgroups to those of existing standards, under the assumption that headgroups are the chemical feature most likely responsible for differences in ionization efficiency among observed IPLs. For instance, Popendorf et al. (2013) found that response factors varied strongly between IPLs of different headgroups and less so from chain length during HPLC electrospray ionization.

Authentic standards and response factors are shown in Table S1 and their assignments to observed IPLs are reported in Table 1. IPLs with headgroup analogs among standards include monoglycose (1G), diglycose (2G), sulfoquinovose (SQ), phosphatidylinositol (PI), diphosphatidyl glycerol (DPG), phosphatidylcholine (PC), phosphatidyl (N,N-dimethyl)ethanolamine (PDME), phosphatidylethanolamine (PE), phosphatidylglycerol (PG), phosphatidyl (N-methyl)ethanolamine (PME), and phosphatidylserine (PS). The rationale guiding response factor assignments for IPLs with no direct headgroup analog are briefly outlined below. IPLs with nitrogen-bearing groups, such as diglycosyl (N-acetyl)glycosaminyl glycose (2G-NAcG-G), triglycosyl (N-acetyl)glycosaminyl glycose (3G-NAcG-G), monoglycosyl (N)glycosamine (G-NG), (N)glycosaminyl glycoronic acid (NG-GA), glycosyl (N-methyl) glycosaminyl glycosyl phosphate (G-MeNG-G-P), glycosyl (N)glycosaminyl glycosyl phosphate (G-NG-G-P), (N-methyl)glycosaminyl glycosyl phosphate (MeNG-G-P), (N-acetyl)glycosaminyl phosphate (NAcG-P), (N)glycosaminyl monoglycosyl phosphate (NG-G-P), aminophosphopentanetetrol (APT), ceramide (CER) lipids, and lipids with an unidentified “223” headgroup (see Figure S4) were assigned the response factor obtained from C42:0 PC diacylglycerol (DAG), under the assumption that the nitrogen-bearing functional groups contained in these lipids might result in comparable ionization efficiencies. This assumption was based on qualitative assessment of the differences in relative peak intensities of nitrogen-bearing and non-nitrogen bearing standards; the former had peak intensities that averaged about an order of magnitude greater than the latter (Table S1). Lipids with a glycoronic acid (GA) headgroup, a non-nitrogen-containing single-moiety glycosyl group, were assigned the response factor obtained from a 1G-DAG standard composed of a mixture of C34:2 and C34:3 chain lengths. Lipids with a monoglycosyl glycoronic acid (G-GA), triglycose (3G), or tetraglycose (4G) headgroup were assigned the response factor of the standard containing the closest number of non-nitrogen-bearing glycosyl moieties; a 2G-DAG standard mixture of C34:2, C34:3, and C36:6 chain lengths. Glycophospholipids with no nitrogen, such as monoglycosyl phosphate (1G-P), diglycosyl phosphate (2G-P) or triglycosyl phosphate (3G-P) headgroups, were assigned the response factor obtained from C32:0 PI-DAG. All GDGTs were assigned the response factor obtained from the 1G-GDGT-PG standard that included a mixture of H-shaped and non-H-shaped alkyl chains with 0-3 internal rings. All aminolipids, including ornithine lipids (OL), monohydroxylated ornithine lipids (OL-OH), trimethylornithine lipids (TM-OL), monohydroxylated trimethylornithine lipids (TM-OL-OH), trimethyllysine lipids (TM-KL) and betaine lipids (BL), were assigned the response factor obtained from C32:0 1,2-dipalmitoyl-sn-glycero-3-O-4′-[*N*,*N*,*N*-trimethyl(d9)]-homoserine (DGTS-d9) based on structural similarities of their amino acid headgroups.

### 2.6. IPL Structural Designations and Chemical Formulae

Headgroups, backbones, and alkyl chains serve as the three basic building blocks comprising IPL structure, each with its own set of observed structural variations. The schematic used to categorize divisions among observed headgroup-backbone-alkyl chain variation is shown in Figure 1, with green, blue, and orange portions indicating headgroup, backbone, and alkyl chain structures, respectively. When considering differences across widely varying lipid structures, it is important to define strict boundaries between components for the sake of consistently comparing properties that depend on chemical formulae, such as Z_C_ or the number of aliphatic carbons in an alkyl chain. This is particularly important for comparing lipids with glycerol backbones to those without. Generalized lipid structures with glycerol backbones considered in this study include diether glycerol (DEG, **1**), mixed acyl/ether glycerol (AEG, **2**), diacyl glycerol (DAG, **3**), GDGT (**4**), DPG (**5**), and putative structures NAcG-P (**6**, DAG variant shown), 2GNAcG-G (**7**, DAG variant shown), and 3GNAcG-G-DEG (Figure S7). Structures that do not contain a glycerol backbone include 1,2-alkanediols (**8**), CER lipids (**9**), fatty acid esters of hydroxy fatty amides (FA-OH-FAm, **10**), and monohydroxylated FA-OH-FAm lipids (FA-OH-FAm-OH, **11**).

The headgroup of an IPL was structurally designated as one or more covalently bonded polar moieties linked to one or more backbones, represented by green structures in Figure 1. In some cases, the headgroup itself may be directly linked to one or more alkyl chains, such as in structures **6** and **7**. The chemical formulae of all headgroups reported in Table 1 are assumed to be protonated to an extent that results in a neutrally-charged IPL, though it should be noted that Z_C_ is not affected by pH-dependent ionization. The +1 charge imparted by a quaternary ammonium functional group is not the result of pH-dependent ionization and therefore affects Z_C_, which is why this charge is included in the chemical formulae of PC, TM-OL, TM-OL-OH, and TM-KL.

Most IPL structures observed in this study have one mole of headgroup per mole of lipid with the exception of GDGTs, which has two. It has been shown that sugar and phosphate moieties can be distributed between the two headgroups of a GDGT, resulting in a variety of possible isomers (Yoshinaga et al., 2011). In this study, we could determine the total number and type of moieties among the two headgroups of GDGTs but the analytical method did not allow us to determine headgroup positions. For instance, the two hexose moieties in 2G-GDGT may be clustered on one end of the lipid or evenly distributed among both ends. Our definition of an IPL headgroup was chosen to ensure consistency in headgroup chemical formulae regardless of position. All configurations of headgroup moieties in 2G-GDGT, for example, have the same total elemental abundance. A 2G-GDGT with two monoglycosyl headgroups, each with the formula C_6_H_11_O_5_, has a total elemental abundance of C_12_H_22_O_10_ among headgroups. If the 2G-GDGT has one diglycosyl headgroup with the formula C_12_H_21_O_10_, then its other headgroup must be a single hydrogen atom to bring the total to C_12_H_22_O_10_. In GDGTs with every headgroup moiety clustered on one side, this hydrogen atom is defined as belonging to the hydroxyl group on the opposite end of the GDGT, and is listed as its own headgroup in Table 1.

**Figure 2:**
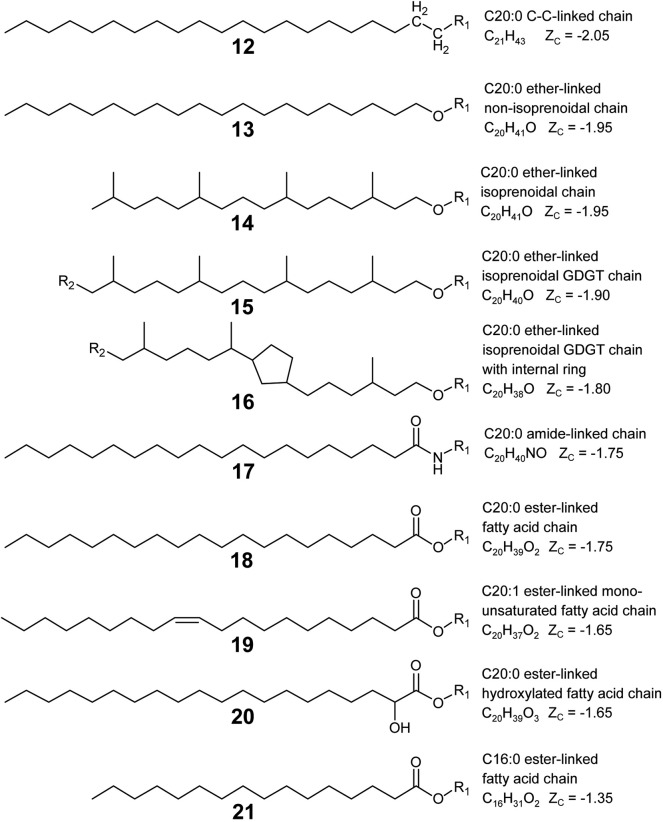
Lipid alkyl chain modifications and backbone-chain linkage types organized by Z_C_ from reduced (top) to oxidized (bottom). Example structures were chosen to permit comparison of Z_C_ between various types of alkyl chain modifications: chain-backbone linkage type as C–C **(12)**, ether **(13)**, amide **(17)**, or ester **(18)**; non-branching and branching chains **(13, 14)**; isoprenoidal non-GDGT chains and GDGT half-chains **(14, 15)**; GDGT half-chains without and with an internal ring **(15, 16)**; saturated and unsaturated chains **(18, 19)**; non-hydroxylated and hydroxylated chains **(18, 20)**; and chains with a greater and lesser number of aliphatic carbons **(18, 21)**. R_1_ represents a covalent bond to the rest of the lipid, and R_2_ indicates a covalent bond with another GDGT half-chain.

Lipid backbones are designated by the blue structures in Figure 1. Most IPLs observed in this study have one mole of backbone per mole of lipid. However, GDGT (**4**) and DPG (**5**) lipids were counted as having two moles of backbone per mole of lipid. IPL backbones were structurally designated according to two criteria chosen to promote consistency among observed IPL structures. First, the backbone must have a linear aliphatic chain of three carbons. Second, the backbone must include three “connector” functional groups that serve to anchor the headgroup and alkyl chains. In addition to these three connector groups, the three-carbon backbone may include modifications like hydroxylations or carbonyl groups. Various backbone-alkyl chain linkage types are shown in Figure 2, with backbone connector groups shown proximal to the R_1_ group representing the rest of the backbone; a methylene group (CH_2_) for a carbon-carbon (C–C) link **(12)**, an oxygen atom (–O–) for an ether link **(13–16)**, –NH– for an amide link **(17)**, or an oxygen atom (–O–) for an ester link **(18–21)**. Connector groups were included in the structure of the backbone rather than in the alkyl chain to maintain consistency when calculating nC in alkyl chains with C-C backbone-chain linkage relative to other chains. To demonstrate, consider the C-C linked alkyl chain **(12)** and the ether-linked alkyl chain **(13)** in Figure 2. Both are saturated and have approximately the same physical length. To ensure that both chains have the same value for nC, the connector groups proximal to R_1_ must be categorized as part of the backbone. Structures **8** through **11** illustrate how the backbone contains one CH_2_ group (in blue) and the alkyl chain contains the other (in orange) in a C-C link.

Alkyl chains are aliphatic hydrocarbon chains linked to the IPL backbone or in some cases, directly to the headgroup. Their designation is indicated by orange structures in Figure 1. Alkyl chains begin at the carbon atom directly after the backbone “connector” atom and continue to the distal methyl group that caps the end of the chain. Most of the IPL structures observed in this study had two moles of alkyl chains per mole of lipid **(1–3, 8–11)**, though some had three **(6–7)** or four **(4–5)**. Each GDGT had two membrane-spanning biphytanyl alkyl chains with forty carbons apiece. GDGT chains were conceptually divided into twenty-carbon half-chains to permit averaging of chain properties, such as Z_C_ and nC, between GDGTs and non-GDGT bilayer lipids. Therefore, one mole of GDGT was counted as having four moles of half-chains (see Structure **4**) so that they could be more directly compared to the alkyl chains of non-GDGTs. GDGT half-chains terminate in a methylene group (CH_2_) that is covalently bonded to the methylene group of another GDGT half-chain within the membrane interior (e.g., Structures **15** or **16**).

### 2.7. Calculation of Average Lipid Properties and Elemental Composition

Abundance-weighted properties of IPL headgroups, backbones, and chains were calculated for each sample using the equation

(2) $$\begin{array}{l} \Xi =\frac{\sum_{i}\Xi_{ipl,i}\cdot x_{i}}{\sum_{i}n_{component,i}\cdot x_{i}}, \end{array}$$

where Ξ indicates the average property of interest (e.g., average nC of IPLs in a sample), Ξ_*ipl, i*_ represents the property summed across all components of the same type in the *i*th IPL (e.g., 32 carbons in the alkyl chains of a C32 IPL) with *n*_*component, i*_ instances of the component in the IPL (e.g., 2 alkyl chains in a DAG IPL). Finally, *x*_*i*_ represents the mole fraction of the *i*th IPL. Abundance-weighted properties calculated in this way include nC, nUnsat, and the number of hydroxylations (nOH) per alkyl chain, the fraction of alkyl half-chains belonging to GDGT (*x*_*GDGT*_), and the fraction backbone-alkyl chain linkage types with an ether (*x*_*ether*_), ester (*x*_*ester*_), amide (*x*_*amide*_), or C-C (*x*_*C*−*C*_) bond. The abundance-weighted number of internal rings per GDGT (not per alkyl chain) in a sample was also calculated using Equation (2) by setting *x*_*i*_ to the mole fraction of the *i*th GDGT (rather than the *i*th IPL), and setting *n*_*component, i*_ equal to one, thereby producing a per-GDGT property and not a per-chain property.

Average chemical formulae of IPLs and their component parts were also calculated for each sample using Equation (2) by substituting Ξ_*ipl, i*_ with charge and elemental abundances of carbon, hydrogen, nitrogen, oxygen, phosphorus, and sulfur atoms in the *i*th IPL. The value of *n*_*component, i*_ was set to one when calculating average chemical formulae of full IPLs. Even when the structure of an IPL or component is unclear, its elemental composition is typically obtainable with high resolution accurate-mass mass spectrometry. This permits the inclusion of ambiguous structures when calculating average chemical formulae. For example, the unidentified “223” headgroup is suspected to have the chemical formula C_7_H_12_NO_6_ based on mass spectral interpretation (Figure S4) and could therefore be included in the calculation of average chemical formulae of IPLs and headgroups.

### 2.8. Calculation of IPL Z_C_

A step-by-step example illustrating how lipid Z_C_ can be calculated for a hypothetical sample is provided in the Supplementary Material. The Z_C_ of IPLs and their component parts were calculated using the equation

(3) $$\begin{array}{l} {\text{Z}}_{\text{C}}\text{=}\frac{2o+3n-5p-4s-h+Z}{c}, \end{array}$$

where *Z* stands for the net charge and *c*, *h*, *n*, *o*, *p*, and *s* represent the number of atoms of carbon, hydrogen, nitrogen, oxygen, phosphorus, and sulfur in the chemical formula of interest. Hydrogen, oxygen, and nitrogen were assigned oxidation states of +1, −2, and −3. Sulfur within the sulfonic acid group of SQ-DAG was assigned an oxidation state of +4. Phosphorus was assigned an oxidation state of +5 to be consistent with that of phosphorus within the phosphate ion. Charge gained or lost by pH-dependent protonation or deprotonation, as is common in many lipid headgroups, does not affect Z_C_.

Equation (3) was used to determine the Z_C_ values of individual lipid structures, such as those reported in Figure 2. It was also used to calculate abundance-weighted Z_C_ values in each sample; in this case, using the abundance-weighted charge and carbon, hydrogen, nitrogen, oxygen, phosphorus, and sulfur atoms in the average chemical formulae of IPLs and their components.

### 2.9. Statistical Simulation of Analytical Uncertainty

We conducted a statistical analysis to check whether observed trends in Z_C_ were not an artifact of the methods chosen for IPL quantification. To do this, we employed an R script to carry out a Monte Carlo-style bootstrap sensitivity analysis in which manually integrated IPL HPLC-MS peak areas were allowed to vary randomly by up to 30% of their original value over 999 iterations. In addition, the analytical response factors applied to any headgroup-backbone combination listed in Table 1 were allowed to vary by up two orders of magnitude higher and lower. Z_C_ for lipids and their components were re-calculated from average chemical formulae after each iteration.

## 3. Results and Discussion

### 3.1. Hot Spring Sample Sites

Temperature, pH, and conductivity measurements are shown in Table 2 for samples taken along the outflow channels of Bison Pool, Mound Spring, Empress Pool, and Octopus Spring. Upstream samples collected closest to the source pools of each spring ranged from circumneutral to alkaline (pH 5.78–8.81), with temperatures close to the boiling point of water (82.2–91.0°C) given their altitude in YNP (~2,200 m above sea level at Bison Pool and Mound Spring, 2,250 m at Octopus Spring, and 2,300 m at Empress Pool). Water temperature decreased and pH increased with distance from the source, with the furthest samples downstream ranging between 29.0 and 59.8°C and pH 8.27 and 9.53. Trends in conductivity were typically non-linear and were not shared among hot springs.

**Table 2:** Selected geochemical and physical data from each sample site.

**Site**	**Sample**	**12T UTM coordinates**		**Dist^**a**^** **(m)**	**Zone^**b**^**	**Temperature** **(^**°**^^C^)**	**pH**	**Conductivity^**c**^** **(μS/cm)**
		**Easting**	**Northing**					
Bison	BP1	510710	4935155	2.9	C	89.0	7.23	1550
Pool	BP2	510715	4935156	8.2	C	80.9	7.34	1568
	BP3	510718	4935157	11.1	T	73.3	7.27	1540
	BP4	510719	4935159	13.4	P	63.1	8.09	−^d^
	BP5	510719	4935163	17.2	P	40.5	8.25	1508
	BP6	510724	4935165	22.6	P	29.0	9.01	1697
Mound	MS1	511114	4934621	3.6	C	91.0	8.81	1612
Spring	MS2	511108	4934624	12.7	C	77.3	8.65	1621
	MS3	511098	4934628	24.2	P	64.8	9.08	1617
	MS4	511083	4934621	38.7	P	53.0	9.22	1634
	MS5	511049	4934625	53	P	35.1	9.53	1660
Empress	EP1	0521589	4948280	2.2	C	82.2	5.78	1824
Pool	EP2	0521585	4948280	6.2	T	70.5	6.96	1832
	EP3	0521580	4948285	13.3	T	60.7	7.63	1840
	EP4	0521560	4948293	34.8	P	51.6	7.99	1860
	EP5	0521558	4948295	37.6	P	38.1	8.42	1664
Octopus	OS1	0516054	4931217	7.0	C	85.4	7.29	1622
Spring	OS2	0516016	4931212	38.3	P	59.8	8.27	1581

a Distance from hot spring source.

b Major metabolic regime representative of the microbial community at the sample site, interpreted visually in the field based on the presence or absence of photosynthetic pigments; C, strictly chemosynthetic; T, transition to phototrophy; P, photosynthetic.

c Conductivity was normalized to 25 °C using the formula Cond_T_/(1+α(T−25)), where Cond_T_ stands for the conductivity measured at the temperature of the sample site and α represents the temperature correction coefficient taken as 0.02 for freshwater.

d *No data*.

Microbial communities inhabiting the sediment below the surface of the water changed visibly downstream. At Bison Pool and Octopus Spring, upstream samples contained white or pink streamer biofilm communities (SBCs) clinging to submerged pebbles or mineral protrusions. A study by Meyer-Dombard et al. (2011) found the bulk of the bacterial community in the pink streamers of Bison Pool belonged to *Aquificales* and *Thermatogales*, while most archaea were mainly comprised of *Crenarchaeota* and *Desulfurococcales*. SBCs were absent at Empress Pool and Mound Spring, despite the latter's proximity and apparent geochemical similarity to Bison Pool. Communities of photosynthetic microorganisms were visually identified in downstream samples by their pigmentation. At Bison Pool, Mound Spring, and Octopus Spring, the transition from chemotrophic to mixed photo/chemo-trophic microbial communities could be readily identified by the sharp onset of photosynthetic pigmentation over a span of a few centimeters. This transition at Empress Pool was not as distinct, and visual confirmation of samples with phototrophic communities relied on faint patches of pigmented microorganisms. The maximum temperature we observed for phototrophic organisms was at 73.3°C at BP3, which falls close to the maximum temperature limit observed for photosynthesis among alkaline YNP hot springs reported by Cox et al. (2011).

Downstream from the “photosynthetic fringe,” laminated green/orange photosynthetic mats were present at Bison Pool, Mound Spring, and Octopus Spring. Previous work by Ward et al. (1987) at Octopus Spring and Meyer-Dombard et al. (2011) at Bison Pool and Mound Spring showed that *Synechococcus* cyanobacteria and *Chloroflexus* bacteria comprise the bulk of the community at these mats. Empress Pool did not have a massive laminar microbial mat, though green/orange photosynthetic communities were visually apparent along either side of the channel in broken patches downstream. Samples BP6 and MS5, taken from post-mat runoff zones of gray-beige flocculent matter, were identified as hosting photosynthetic microbes based on visual confirmation of green pigmentation within the floc.

Microbial mats like those found at Bison Pool, Mound Spring, and Octopus Spring are complex stratified ecosystems of interconnected metabolic cycles. The oxic conditions measured in the water column and upper mat give way to anoxia after only a few millimeters depth (Dupraz and Visscher, 2005; Franks and Stolz, 2009). Further, redox conditions in a microbial mat have been shown to undergo extreme fluctuations throughout a 24-h period (Fenchel, 1998; Visscher et al., 1998; Jonkers et al., 2003). The upper few millimeters of a mat are supersaturated with O_2_ from cyanobacterial photosynthesis during the day and anoxic within minutes to hours of darkness. In this study, we strove to minimize the influence of sunlight availability, diel cycles, and other complicating factors arising from the passage of time by collecting samples at each outflow channel during light hours of the same day. In this way, we sought to capture a snapshot of water chemistry and lipid profiles from which to draw broad correlations. There is an intriguing possibility of steep biogeochemical gradients coinciding with substantial changes in lipid composition across a depth of millimeters, though these would have been interpreted as bulk averages according to our sampling method.

### 3.2. Water Chemistry

**Figure 3:**
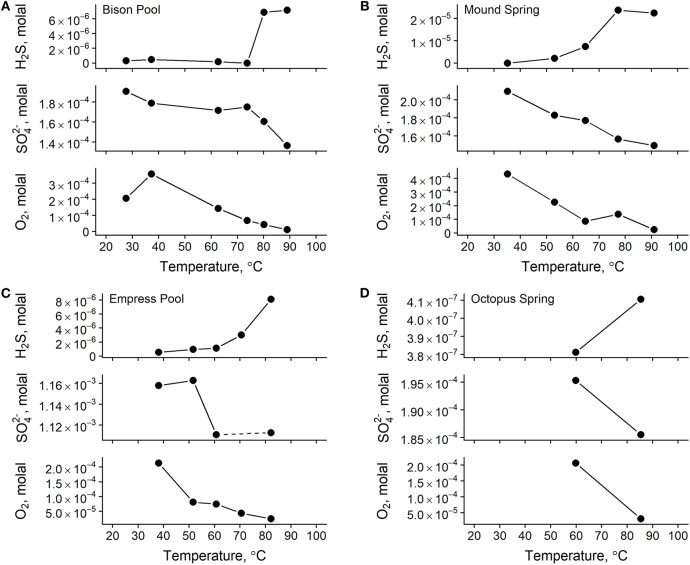
Total concentrations of redox-sensitive aqueous chemical species in samples from Bison Pool **(A)**, Mound Spring **(B)**, Empress Pool **(C)**, and Octopus Spring **(D)**. Lines between points are meant to guide the eye between measurements only. A water sample was not collected for sulfate at Empress Pool site EP2 during the 2012 field season, indicated here by a dashed line between sulfate measurements for sites EP1 and EP3.

Measured concentrations of dissolved oxygen, nitrate, nitrite, sulfate, total ammonia, and total sulfide are given in Table 3. In all four outflow channels, concentrations of dissolved oxygen were lowest in samples closest to the source and increased downstream. This could be attributable to a combination of factors, such as extent of mixing with atmospheric O_2_, input from microbial oxygenic photosynthesis, and concentration via evaporation. Sulfate concentrations also increased downstream and coincided with decreasing sulfide concentrations, as shown in Figure 3. A previous experiment by Cox et al. (2011) concluded that abiotic processes, such as degassing, oxidation by O_2_ and hydrogen peroxide, and mineral precipitation were too slow to explain the downstream decrease in sulfide concentration at Bison Pool, and that oxidation of sulfide by outflow channel microorganisms was likely responsible. It is feasible that biological oxidation of sulfide may also be occurring in the outflow channels of Mound Spring, Empress Pool, and Octopus Spring given the parallels in geochemistry observed among these springs. In addition to sulfide and sulfate, we observed an inverse correlation between ammonia and nitrate concentrations, with the highest concentrations of ammonia closest to the source and decreasing downstream while nitrate concentrations steadily increase (Table 3). Oxidation of ammonia into nitrite and nitrate by microbial communities via nitrification is one possible explanation. However, previous phylogenetic and metagenomic studies of nitrogen-cycling genes in Bison Pool and Mound Spring microbial communities have found either an absence of genes involved in ammonia oxidation (Swingley et al., 2012), or limited presence with no active expression (Loiacono, 2013). Regardless of the cause, depletion of sulfide and ammonia coinciding with an increase in sulfate, nitrate, and dissolved oxygen suggests that water is most reduced at the source and becomes increasingly oxidized downstream along these hot spring outflow channels. This would agree with previous oxidation-reduction potential (ORP) Ag/AgCl electrode measurements that confirmed a downstream increase in Eh along the outflow channels of Bison Pool and Mound Spring (Dick and Shock, 2011).

**Table 3:** Concentrations of selected redox-sensitive dissolved chemical species^a^.

**Site**	**Sample**	**Oxidized**				**Reduced**	
		**O_2_**	**NO_3_^−^**	**NO_2_^−^**	****∑**SO_4_^2−^**	****∑**NH_4_^+^**	****∑**HS^−^**
		**(mg l^−1^)**	**(mg l^−1^)**	**(mg l^−1^)**	**(mg l^−1^)**	**(mg l^−1^)**	**(μg l^−1^)**
Bison	BP1	0.2	0.01	0.02	13.11	0.07	230
Pool	BP2	0.7	0.01	0.04	15.43	0.06	220
	BP3	1.1	0.02	0.01	16.81	0.04	bdl^b^
	BP4	2.3	0.03	0.02	16.50	0.02	6
	BP5	5.7	0.004	bdl	17.18	0.01	15
	BP6	3.3	0.07	bdl	18.32	0.02	10
Mound	MS1	0.4	0.01	bdl	14.33	0.07	716
Spring	MS2	2.2	0.01	bdl	15.03	0.01	758
	MS3	1.4	0.04	0.02	16.99	0.03	236
	MS4	3.6	0.02	0.01	17.56	0.02	70
	MS5	6.9	0.06	bdl	20.11	bdl	bdl
Empress	EP1	0.4	0.01	0.08	106.87	0.42	260
Pool	EP2	0.7	–^c^	–	–	–	97
	EP3	1.2	0.01	bdl	106.70	0.31	37
	EP4	1.3	0.03	0.03	111.70	0.39	31
	EP5	3.4	0.08	0.01	111.24	0.14	18
Octopus	OS1	0.5	0.03	0.03	17.82	0.06	13
Spring	OS2	3.3	0.03	0.02	18.76	0.02	12

a Sulfide (HS^−^), ammonium (NH_4_^+^), and sulfate (SO_4_^2−^) concentrations are summed for their respective pH-dependent protonated states.

b bdl: below detection limit.

c *No data*.

### 3.3. IPL Headgroup and Backbone Distributions

**Figure 4:**
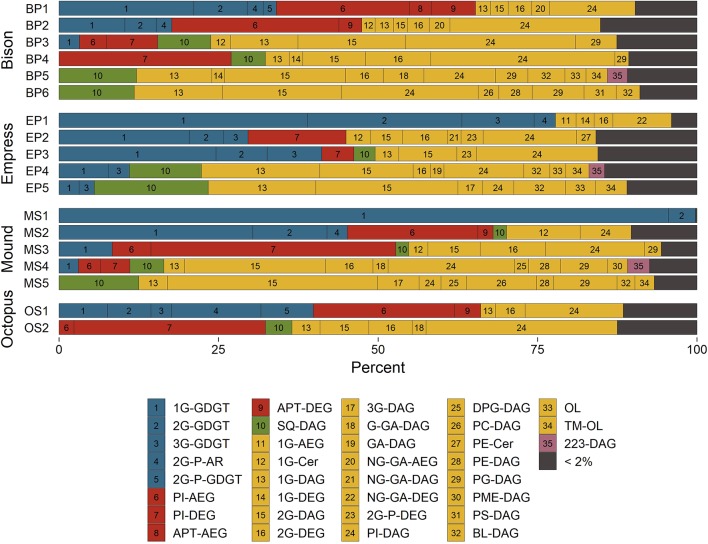
Distributions of hot spring microbial IPLs classified by their headgroup-backbone-chain linkage. See text for abbreviations. Bar numbers reference indices in the legend. Bar colors represent suspected source organisms; unspecific Archaea (blue), *Aquificales* (red), phototrophs (green), unspecific Bacteria and Eukarya (yellow), and unknown (purple).

Mole fractions of IPLs grouped according to their headgroup/backbone combinations and color-coded by their suspected source organisms are shown in Figure 4. Archaeally-derived GDGT and archaeol (AR) lipids (Figure 4, blue bars 1–5) were most abundant in samples collected closer to the source of each hot spring. MS1 and EP1 were the only two samples where archaeal lipids outnumbered those of non-archaea. Considering that one mole of GDGT is functionally equivalent to two moles of non-GDGTs in a membrane bilayer (in a general sense), it is unsurprising that archaeal membranes comprise the majority of lipids in the hottest upstream samples where Archaea are expected to thrive (Barns et al., 1994, 1996; Meyer-Dombard et al., 2005; Schouten et al., 2007b; Zhang et al., 2008). 1G-GDGT was found in >2% abundance in all but five samples and was always more abundant than any other archaeal lipid except for 2G-P-AR in sample OS1. Upstream samples were typically rich in lipids thought to be diagnostic of members of *Aquificae* bacteria (Figure 4, red bars 6–9), including PI-DEG/AEG and APT-DEG/AEG (Sturt et al., 2004; Schubotz et al., 2013). Indeed, previous phylogenetic analyses have confirmed the presence of *Aquificae* in the SBCs of Bison Pool and Octopus Spring, and in the high-temperature sediments of Mound Spring, and Empress Pool (Reysenbach et al., 1994; Meyer-Dombard et al., 2005, 2011; Spear et al., 2005; Swingley et al., 2012; Schubotz et al., 2013; Beam et al., 2016; Colman et al., 2016; Romero, 2018). SQ-DAG lipids found in the photosynthetic membranes of cyanobacteria and other phototrophs (Sato, 2004) were located in mid-to-downstream samples where green/orange pigments were visually confirmed in microbial communities (Figure 4, green bar 10). These sites also hosted an abundance of other lipids common in, but not specific to, phototrophic membranes, such as DAG lipids with 1G, 2G, and PG headgroups (Murata and Siegenthaler, 1998).

Glycolipids were abundant in every sample (Figure 4, yellow bars 11–22), with mole fractions ranging from 31% in OS2 to nearly 100% in MS1. Glycophospholipids (Figure 4, yellow bars 23–24), especially PI-DAG, were also abundant in nearly every sample and reached up to 63% in OS2. This preponderance of glyco(phospho)lipids is thought to confer heat tolerance via inter-lipid hydrogen bonding (Curatolo, 1987). Evidence to support this hypothesis comes from experiments by Adams et al. (1971), Ray et al. (1971), and Prado et al. (1988) demonstrating that higher growth temperatures result in a greater proportion of glycolipids to other membrane lipids in various thermophilic microorganisms.

Downstream samples collected from Bison Pool, Mound Spring, and Empress Pool featured many DAG phospholipids common to unspecific Bacteria and Eukaryotes (Figure 4, yellow bars 25–31), such as DPG, PC, PE, PG, PME, and PS (Kent, 1995; López-Lara and Geiger, 2017). These lipids were not abundant in Octopus Spring sample OS2, possibly because the temperature measured at this sample site, 59.8°C, roughly corresponds to the upper temperature limit of about 60°C reported for Eukaryotes (Tansey and Brock, 1972). Aminolipids (Figure 4, yellow bars 32–34) were less abundant upstream (typically <2%) than in downstream samples, where they reached up to 19% in sample EP5. IPLs bearing a headgroup with an exact mass consistent with the formula of C_7_H_12_NO_6_, referred to here as “223-DAG” (Figure 4, purple bar 35), were most abundant (2–3%) in samples collected between 40 and 53°C. The structure and source of this lipid is unknown.

### 3.4. IPL Alkyl Chain Properties

Changes in abundance-weighted average properties of microbial IPL alkyl chains were observed downstream in all four outflow channels (Table S2). Patterns in chain-backbone linkage, aliphatic carbon number, degree of unsaturation and GDGT cyclization, and chain hydroxylations are described below.

#### 3.4.1. Chain-Backbone Linkage

**Figure 5:**
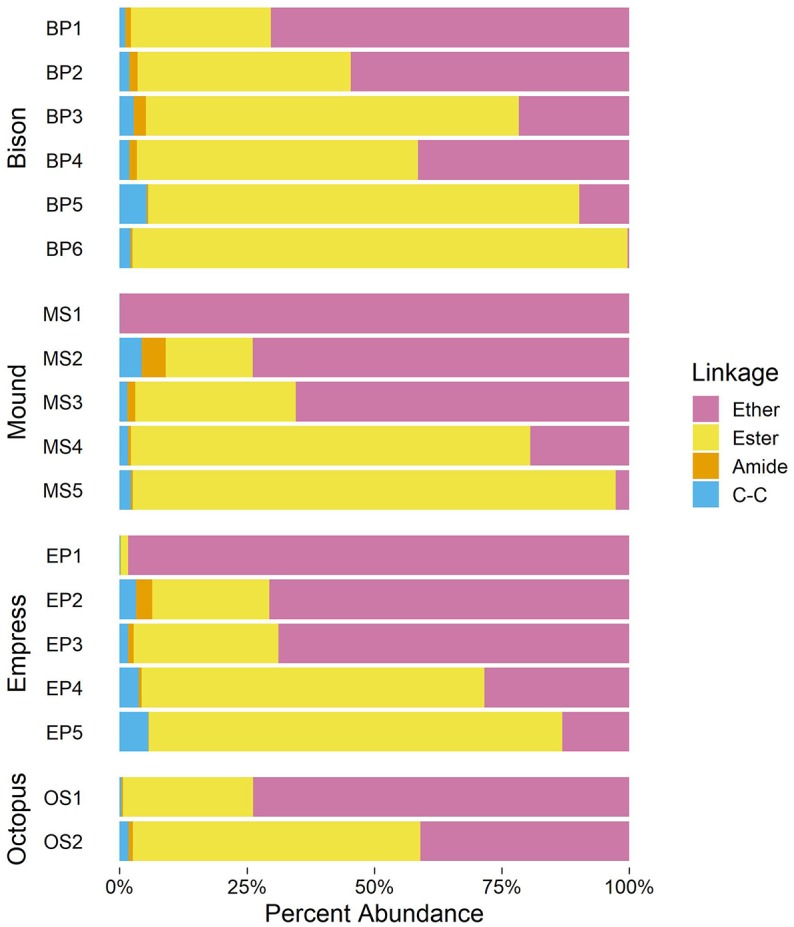
Relative abundances of major backbone-alkyl chain linkage types in samples.

As shown in Figure 5, upstream samples from all four hot spring outflow channels are dominated by ether-linked alkyl chains (70% mole fraction at BP1 to nearly 100% at MS1). Ether-linked alkyl chains are resistant to hydrolysis (Daniel and Cowan, 2000). The proportion of ether-linked chains decreases downstream from each hot spring source, giving rise to a growing proportion of ester-linked chains. The onset of photosynthetic communities coincides with a sharp increase in the proportion of ester-linked chains, comprising over 50% of alkyl chains in all samples containing visible photosynthetic pigmentation. Downstream samples have the highest abundance of ester-linked chains in each outflow channel (81–97% in samples below 40°C). Chains linked to the backbone via an amide or C–C bond are also present, though in relatively low abundance (<5% in any sample).

While ether lipids are the common chain-backbone linkage in archaea, they are a rare occurrence in bacteria. Bacterial ether lipids are predominantly reported from thermophilic bacteria (Huber et al., 1992; Jahnke et al., 2001). However, few mesophilic organisms are also able to synthesize these lipids in culture (Vinçon-Laugier et al., 2017). Contrasting these sparse culturing reports, bacterial ether lipids are abundantly found in the environment, typically under anoxic conditions, such as stratified water columns and marine sediments (Schubotz et al., 2009; Schröder, 2015; Evans et al., 2017). Based on these reports, temperature is not the only environmental variable influencing the distribution of bacterial ether lipids. Similarly, it is unclear whether pH plays an important factor. Some environments, such as marine sediments dominated by the process of anaerobic oxidation of methane where bacterial ether lipids are abundantly found, are more alkaline than their surrounding environment. However, the sediments and stratified water columns in these studies are relatively circumneutral. We therefore explore whether apart from temperature or pH, other variables, such as redox may influence backbone-chain linkage.

#### 3.4.2. Number of Aliphatic Carbons, nC

**Figure 6:**
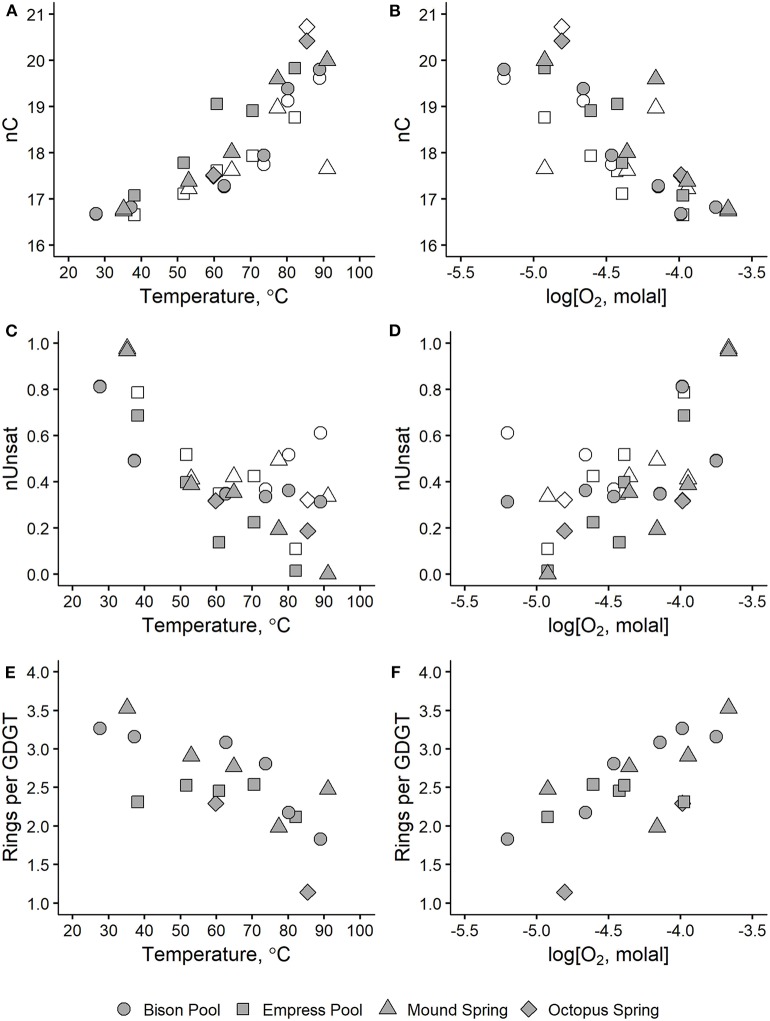
Average alkyl chain properties in hot spring microbial IPLs plotted against temperature (left panels) and dissolved oxygen concentration (right panels) for number of aliphatic carbons, nC **(A,B)**, degree of unsaturation, nUnsat **(C,D)**, and internal rings per GDGT **(E,F)**. Shaded symbols indicate values that include all IPL alkyl chains. Values represented by empty symbols are calculated in the same way but exclude contributions from GDGTs.

As shown in Figures 6A,B, nC of alkyl chains tends to be greatest at the highest measured temperatures (above 80°C) and lowest dissolved oxygen concentration (around −5.0 log molal O_2_). Values of nC approach 20 aliphatic carbons in samples furthest upstream, partly because these samples are rich in GDGTs with four 20-carbon half-chains. However, values of average nC approach 20 aliphatic carbons in upstream samples even after excluding contributions from GDGTs (empty symbols, Figures 6A,B). Sample MS1 represents the only upstream sample where the weighted nC non-GDGT alkyl chains is anomalously small at 17.4 carbons, though it should be noted that non-GDGT chains comprise <1% of alkyl chains in this sample. Sample OS1 has the greatest weighted nC at 20.42 carbons because it was rich in C_50_ and C_55_ 2G-P-AR lipids that averaged 25 or 27.5 carbons per chain. Alkyl chain nC decreased with progressively cooler temperatures and oxidized conditions, reaching about 16.7–17 aliphatic carbons in samples furthest downstream. Overall, these trends in weighted nC agree with a plethora of studies demonstrating the capacity of microorganisms to adapt their membrane fluidity and permeability in response to temperature by adjusting the strength of hydrophobic interactions in the non-polar portion of their membranes (see reviews by Denich et al., 2003; van Meer et al., 2008; Siliakus et al., 2017).

#### 3.4.3. Degree of Unsaturation, nUnsat

Average degree of unsaturation in IPL alkyl chains increased downstream in all four outflow channels, as shown in Figures 6C,D. Samples above 50°C or below -4.0 log molal O_2_ had values of weighted nUnsat between 0 and 0.4 unsaturations and increased to 0.4–1 unsaturations in samples further downstream. This trend is especially prominent at Mound Spring and Empress Pool, where nUnsat is near-zero in MS1 and EP1 and gradually increases to about 0.7 and 1 unsaturation per chain in MS5 and EP5, respectively. Inverse correlations between temperature and average degree of unsaturation are well-documented in the literature (Marr and Ingraham, 1962; Siliakus et al., 2017). An unsaturation can either be in the *cis* and *trans* configuration; a *cis*-unsaturation is thought to increase membrane fluidity by introducing a “kink” in an alkyl chain that decreases chain packing and disrupts neighboring lipids in the membrane. Conversely, relatively straight *trans*-unsaturated fatty acids reduce membrane fluidity and have been correlated with microbial growth at higher temperatures and resistance to solvents and desiccation (Okuyama et al., 1990, 1991; Weber et al., 1994; Halverson and Firestone, 2000; Kiran et al., 2004, 2005). Determination of *cis* or *trans* configurations in unsaturated IPL alkyl chains was beyond the analytical scope of this study, though it should be emphasized that Z_C_ is not affected by isomerism.

#### 3.4.4. Degree of GDGT Cyclization

The incorporation of one or more cycloalkyl rings into the alkyl chains of GDGTs (e.g., Structure **16**) has been proposed to enhance lipid packing while increasing fluidity in archaeal membranes (Gliozzi et al., 1983; Gabriel and Chong, 2000; Chong et al., 2012; Sollich et al., 2017). We observed a downstream increase in the number of internal rings in the GDGTs in the outflow channels of Bison Pool, Mound Spring, and Octopus Spring, as shown in Figures 6E,F. Between samples collected closest to each hot spring source and those furthest downstream, the average number of rings in GDGTs increased from 1.8 to 3.3 between BP1 and BP6, 2.5 to 3.5 between MS1 and MS5, and 1.1 to 2.5 between OS1 and OS2. Samples collected from the outflow channel of Empress Pool averaged between 2.1 and 2.5 rings and showed no clear correlation with temperature or dissolved oxygen concentration.

This downstream increase in the number of rings per GDGT agrees with a previous study by Schubotz et al. (2013) reporting a similar trend along the Bison Pool outflow channel. However, these results do not corroborate with studies by Schouten et al. (2002, 2007a) that propose a positive correlation between GDGT chain cyclization and temperature as the basis for the TEX_86_ paleothermometer (though TEX_86_ was originally calibrated for sea surface temperatures). Additionally, these observations do not match the results of laboratory growth experiments demonstrating increased chain cyclization with temperature in thermoacidophilic archaea (Boyd et al., 2011), marine *Crenarchaeota* (Wuchter et al., 2004), and *Thaumarchaeota* (Elling et al., 2015). Conflicting trends have also been reported in terrestrial hydrothermal systems. Kaur et al. (2015) observed an increase in GDGT ring abundance with temperature between a pH range of 5.5–7.2 in hot springs from the Taupo volcanic zone in New Zealand, though their low pH samples did not. Wu et al. (2013) studied GDGTs in Yunnan hot springs, China, and found that ring index increased with temperature in one statistical grouping and increased with acidity in the other.

Low pH has been observed to cause an increase in GDGT chain cyclization (Macalady et al., 2004; Boyd et al., 2013), which has been attributed to a denser packing of the cell membrane resulting in a higher tolerance toward large ion gradients (Gabriel and Chong, 2000). Studies on the influence of high pH on the incorporation of the number of rings, comparable to our systems, have not been systematically conducted, therefore it remains unclear whether the change in pH from alkaline to circumneutral plays an important role in the downstream increase in the number of rings. However, we find it conversely conceivable that the lower pH conditions upstream may cause the incorporation of more rings for a denser packing of the membrane, which is not reflected in our data. Temperature and pH have been cited as competing variables in numerous studies of GDGT ring index in hydrothermal systems and thermophilic archaea (Pearson et al., 2008; Boyd et al., 2011, 2013). It is still unclear how combinations of geochemical variables influence the incorporation of rings into archaeal membranes in a predictable way, though we propose that environmental redox conditions may influence ring distributions in section 3.6.

#### 3.4.5. Number of Hydroxylations, nOH

Alkyl chains bearing a secondary hydroxyl group (e.g., Structure **20**) comprised only a small portion (<1%) of total chains in our sample set (see Table S2). Even if the backbone hydroxylation of CER (**9**) and FA-OH-FAm-OH (**11**) are counted toward chain nOH, the total proportion of hydroxylated chains would only increase to a maximum of 4% in any sample.

### 3.5. Z_C_ of IPLs and Their Components

Downstream changes in IPLs and their component parts are reflected in their average chemical formulae (Table S3) and consequently, their Z_C_ values (Table S4). Trends in the Z_C_ of full IPLs and their headgroups, backbones, and alkyl chains are described below.

#### 3.5.1. Full IPLs

**Figure 7:**
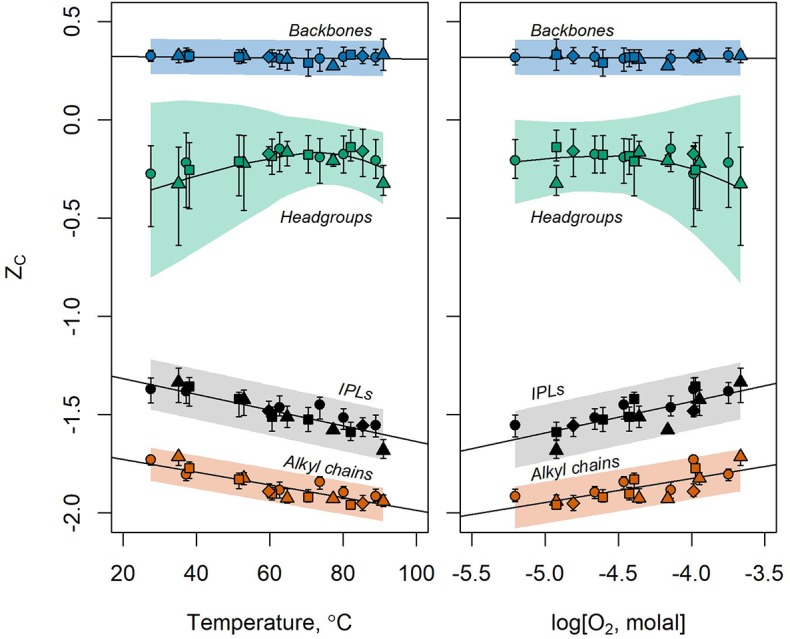
Z_C_ of IPLs (black) and their headgroups (green), backbones (blue), alkyl chains (orange) sampled along the outflow channels of Bison Pool (circles), Mound Spring (triangles), Empress Pool (squares), and Octopus Spring (diamonds) with respect to temperature (left) and log molality of dissolved O_2_ (right). Symbols designate the observed values of Z_C_ of extracted lipids and their components. Bars around the points show the standard deviation of 999 Z_C_ values resulting from the random variation of analytical peak areas and response factors during the bootstrap sensitivity analysis. Regression of these bootstrap values are indicated by fitted lines. Full lipids, backbones, and alkyl chains are fitted with linear regressions while headgroups are fit by local polynomial regression (LOESS). Shaded areas represent 95% prediction intervals for values of Z_C_ produced by the sensitivity analysis. LOESS regression was performed in R using the loess.sd function (“msir” package version 1.3.2) with parameters nsigma = 1.96 (for the 95% prediction interval) and span = 0.9 (for smoothing).

Abundance-weighted Z_C_ values calculated for microbial IPLs collected from Bison Pool, Mound Spring, Empress Pool, and Octopus Spring are plotted against temperature and dissolved oxygen concentrations in Figure 7. It can be seen that decreasing temperature and increasingly oxidized conditions coincide with near-linear changes in the Z_C_ of IPLs. Carbon was most reduced in IPLs sampled closest to each hot spring source (Z_C_ between −1.68 and −1.56 in samples BP1, MS1, EP1, and OS1) where temperatures were highest (82.2–91.0°C) and oxidized inorganic species (nitrate, sulfate, and oxygen) had the lowest concentrations. In progressively downstream samples, carbon in IPLs became more oxidized, with Z_C_ between −1.36 and −1.33 for samples in between 29.0 and 38.1°C where measured concentrations of sulfate, nitrate, and oxygen tended to be highest.

Downstream trends remain intact after performing a statistical simulation of potential sources of analytical error, as shown in Figure 7 by the black bars above and below the calculated values of Z_C_. These bars indicate the standard deviation of Z_C_ values resulting from 999 iterations of the bootstrap sensitivity analysis. The standard deviations of IPL Z_C_ show little overlap between upstream and downstream samples after random variation of up to 30% for integrated HPLC-MS peak areas and up to two orders of magnitude for response factors.

#### 3.5.2. Headgroups

Z_C_ values of IPL headgroups were most positive in mid-stream samples and most negative in samples furthest upstream and downstream (Figure 7). Upstream samples had relatively reduced headgroup carbon due to an abundance of 1G-GDGT lipids. These lipids have two headgroups according to our structural division scheme; a hexose and a hydrogen atom (e.g., the GDGT in Figure S9). This “hydrogen-only” headgroup drives down the Z_C_ of headgroups in upstream samples where 1G-GDGT is abundant. In downstream samples, phospholipids and aminolipids with relatively reduced headgroup carbon, such as PC, PE, PG, and TM-OL became more abundant and drove down the Z_C_.

Glycolipid headgroups 1G (Z_C_ = $-0.16\bar{6}$) and 2G (Z_C_ = $-0.08\bar{3}$) were among the most abundant headgroups observed in all four springs regardless of temperature or redox state, though they were most abundant, along with the glycolipid SQ (Z_C_ = $-0.16\bar{6}$) in samples where photosynthetic microorganisms are visually apparent. This agrees with observations that 1G, 2G, and SQ glycolipids are abundant in cyanobacterial (Wada and Murata, 2009) and algal (Guschina and Harwood, 2006) photosynthetic membranes.

Phosphate-bearing phospholipids and glycophospholipids, especially with PI headgroups, were more abundant downstream than upstream. However, the incorporation of phosphate itself into headgroups has no effect on abundance-weighted headgroup Z_C_, as there is no difference in Z_C_ between analogous non-phosphorylated and phosphorylated headgroups (e.g., between 1G-P and 1G, or between 2G-P and 2G, *etc*.). The greatest abundance of lipids with an APT phospholipid headgroup (Z_C_ = −0.6) was observed in “pink streamer” thermophile communities sampled from the upstream chemosynthetic zones of Bison Pool and Octopus Spring; this result agrees with previous reports that this lipid is common in streamers with *Aquificales* bacteria (Sturt et al., 2004; Schubotz et al., 2013). However, after applying response factors, the abundance APT was low relative to other headgroups, even in streamer samples.

Values of Z_C_ for headgroups were substantially more sensitive to simulated sources of analytical uncertainty than those calculated for alkyl chains, backbones, or full IPLs. After 999 iterations of randomly adjusting response factors and HPLC-MS peak areas, the standard deviations in Z_C_ values (vertical bars in Figure 7) and the 95% prediction interval for future bootstrap calculations (shaded region) were greatest in magnitude for headgroups and overlapped for most samples. This calls into question the significance of downstream trends in headgroup Z_C_, as they may be mere artifacts of the analytical method we used to quantify lipids.

#### 3.5.3. Backbones

Backbones had the most oxidized carbon of any component owing to a high ratio of electronegative atoms (e.g., oxygen, nitrogen) to carbon. The Z_C_ of IPL backbones did not change significantly with temperature or redox state, as most observed backbones were composed of glycerol regardless of the sample location. A fully-linked glycerol backbone has a chemical formula of C_3_H_5_O_3_ that corresponds to a Z_C_ of $0.33\bar{3}$. This is close to the average value in every sample, with little variation.

#### 3.5.4. Alkyl Chains

Alkyl chains tend to have Z_C_ values ~0.3–0.4 more negative than those of the full structure regardless of the sample. The Z_C_ values of alkyl chains were closest to those of the full lipid compared to either headgroups or backbones, indicating that alkyl chains are the main contributor to the oxidation state of carbon in full lipids. This is unsurprising, given that alkyl chains generally contain more carbon than any other IPL component. Since alkyl chains have a high hydrogen:carbon ratio to facilitate their hydrophobicity, the weighted Z_C_ of IPLs and their chains are significantly more negative than those of headgroups or backbones. Because alkyl chains contribute more to the average oxidation state of carbon in the full lipid than any other component, changes in alkyl chains are most responsible for the correlations observed between Z_C_ and temperature or dissolved oxygen in these hot spring outflow channels. Alkyl chain modifications with the most influence on Z_C_ were backbone-alkyl chain linkage chemistry, nC, nUnsat, and the number of internal rings per GDGT.

Changes in chain-backbone linkage chemistry correspond to a downstream increase in the Z_C_ of microbial IPLs stemming from the difference in chemical formulae between an ester and an ether bond. Carbon is more oxidized in an ester bond relative to an ether because it contains two fewer hydrogens and one more oxygen atom (compare Structures **13** and **18**). The number of aliphatic carbons in alkyl chains also has a great effect on Z_C_. Each additional methylene group increases the chemical formula of a lipid by CH_2_ for saturated chains, or by C_2_H_4_ for each methyl branch. Both of these groups have Z_C_ equal to -2, so as more aliphatic carbons are added, the Z_C_ of an alkyl chain is driven closer to this value. This effect can be seen in Figure 7, where the Z_C_ of alkyl chains approach -2 in upstream samples where average nC is highest. Unsaturated alkyl chains are more oxidized than their saturated counterparts. This is because an alkyl chain with a double bond has two fewer hydrogen atoms (e.g., compare Structures **19** and **18**). This is also true for a GDGT ring (e.g., compare Structures **15** and **16**). As such, increased degree of unsaturation and GDGT ring indices contributed to the downstream increase in Z_C_ for lipids and their alkyl chains. Other alkyl chain modifications did not notably impact Z_C_, either because they were observed at very low abundance, such as chain hydroxylations, or did not substantially change lipid chemical formulae, such as the single hydrogen difference between GDGT half-chains and the isoprenoidal chains of non-GDGTs (e.g., Structure **15** compared to **14**).

As shown in Figure 7, trends involving Z_C_ are more resistant to simulated sources of analytical uncertainty in alkyl chains than in full lipids. Linear regression of Z_C_ values produced by the bootstrap sensitivity analysis results in a narrower 95% prediction interval band for alkyl chains than for full lipids. Further, the standard deviations for individual samples tend to have narrower ranges, and overlap less, in alkyl chains. Taken together, we interpret this as evidence that the downstream increase in the Z_C_ of alkyl chains is not an artifact of the analytical method used to quantify lipid abundance.

### 3.6. Redox and Lipid Composition

Thermodynamic analyses are needed to test how redox constraints influence the energetic favorability of lipid modifications, such as alkyl chain length, degree of unsaturation, ester and ether alkyl chain linkage, headgroup type, and so on. Several experiments have already yielded intriguing evidence that the expression of ringed GDGT alkyl chains is favorable under oxidized conditions. Following a laboratory study by Qin et al. (2015) that implicated O_2_ limitation as a confounding factor when interpreting TEX_86_-derived temperatures, Hurley et al. (2016) showed an inverse correlation between ammonia oxidation rate and degree of ring cyclization in GDGTs of cultured ammonia oxidizing *Thaumarchaeota* and hypothesized that the supply of NH_4_^+^, provides the reduction potential necessary to drive saturation to GDGT-0 during lipid synthesis. Further evidence was provided by (Evans et al., 2018), who showed that ammonia oxidizing *Nitrosopumilus maritimus* produces GDGTs with fewer rings when grown in media containing excess NH_4_^+^. Zhou et al. (2019) found that GDGT cyclization from electron supply limitation also occurred in thermoacidophilic *Sulfolobus acidocaldarius* cultures. If sufficient reduction potential is necessary to saturate GDGTs, then an increasingly limited electron supply from ammonia, sulfide, and/or other electron donors may be responsible for the downstream increase in the average number of rings per GDGT found in Bison Pool, Mound Spring, and Octopus Spring. This may also explain why fewer GDGT rings were observed downstream in this study and in Schubotz et al. (2013), which is seemingly at odds with temperature-derived trends reported in various culture experiments (Wuchter et al., 2004; Boyd et al., 2011; Elling et al., 2015) and used in the TEX_86_ paleothermometer (Schouten et al., 2002, 2007a), though redox conditions were not the focus of these studies.

It is intriguing to consider that other lipid structural modifications, or even entire lipid compositions, may be sensitive to redox constraints. Lipid compositions produced by a microbial community might tend to be “electron-rich” (low Z_C_) under reduced environmental conditions where electron supply is high, and “electron-poor” (high Z_C_) under oxidized conditions where electron supply is low. If such a compositional advantage exists, then trends in lipid Z_C_ may be evident along other environmental redox gradients. For instance, studies of IPL distributions in the Black Sea have reported changes in alkyl chain structure along relatively isothermal redox gradients similar to those found along the hot spring outflow channels in this study; deeper sampling in the water column coincides with a pronounced shift in ester- to ether- linked alkyl chains, an increase in nC, a decrease in nUnsat, and an increase in the abundance of GDGTs and ARs (Schubotz et al., 2009; Schröder, 2015; Evans et al., 2017). Based on our approach, IPLs in the oxic zone of the Black Sea have relatively oxidized carbon, while deeper and more reduced conditions correspond to increasingly reduced carbon. This fits the general pattern observed in this study, and may indicate a biological drive for microbial communities to synthesize stable membranes within the redox constraints of their surroundings. If so, Z_C_ information preserved in lipid biomarker compositions could offer valuable insights into the geochemical paleoredox conditions experienced by source organisms. These hypotheses can be tested in a variety of natural systems by comparing the Z_C_ of IPLs produced by the microbial communities to concurrent redox measurements. Studies that strive for completeness and quantitative accuracy in their underlying lipid and geochemical datasets will be extraordinarily valuable for investigating these potential relationships.

## 4. Concluding Remarks

Lipids were extracted from microbial communities sampled spatially along the outflow channels of four alkaline hot springs in Yellowstone National Park. Chemical formulae and relative abundances of lipid structures interpreted from HPLC-MS/MS were used to calculate the average oxidation state of carbon, Z_C_, for lipids in each sample. Values of Z_C_ were also calculated for lipid headgroups, backbones, and alkyl chains. We found that lipids extracted from microbial communities living under the hottest, most reduced conditions had the most reduced carbon (lowest Z_C_). This is because lipids in upstream samples contained a greater number of modifications that increased their hydrogen-to-carbon ratio, including alkyl chains with a greater number of aliphatic carbons, fewer unsaturations, fewer GDGT rings, and linked to the backbone with a higher proportion of ether bonds. The Z_C_ of lipids increased (representing increasingly more oxidized carbon) with distance downstream, coinciding with more oxidized conditions and cooler temperatures. Lipids sampled furthest downstream had the most oxidized carbon (highest Z_C_) resulting from an abundance of alkyl chain modifications that decreased their hydrogen-to-carbon ratio (fewer aliphatic carbons, higher degree of unsaturation, a greater number of GDGT rings, and a greater proportion of ester-linkage). These alkyl chain modifications permit membrane function in the microbial communities sampled across the extreme temperature gradients of Yellowstone outflow channels. The tendency to find oxidized lipid modifications under oxidized conditions and vice versa for reduced conditions may represent adaptation to limitations in available reduction potential imposed by concentrations of electron donors and acceptors in the surrounding water. If this pattern is widespread, the Z_C_ of bulk lipid compositions could become a useful metric for assessing prevailing redox across a variety of natural settings. Preserved in lipid biomarkers, these Z_C_ signatures could offer a window into redox conditions of the past.

## Data Availability Statement

The code and IPL abundance data used in this study can be found in the repository PolarLipidZC, https://gitlab.com/gmboyer/polarlipidzc.

## Author Contributions

GB and ES conceived the study. GB and JW performed the lipid extractions. GB and FS analyzed the HPLC-MS data. GB performed the calculations. All authors contributed to the writing and revision of this manuscript.

### Conflict of Interest

The authors declare that the research was conducted in the absence of any commercial or financial relationships that could be construed as a potential conflict of interest.
