# Supplementary material for: Carbon Oxidation State in Microbial Polar Lipids Suggests Adaptation to Hot Spring Temperature and Redox Gradients
Source: Front Microbiol. 2020 Feb 20;11:229. doi: 10.3389/fmicb.2020.00229 (PMC7044123; doi:10.3389/fmicb.2020.00229)
Supplement: Supplementary file 1 [file Presentation_1.pdf]

Supplementary Material

1 SUPPLEMENTARY DATA

Analytical response factors.

Table S1 provides information about response factors for the suite of intact polar lipid (IPL) standards used in this work. Response factors were interpreted as the linear slopes of high-performance liquid chromatography-mass spectrometry (HPLC-MS) integrated peak area as a function of injected mass with a y-intercept of zero. The squared correlation coefficient, R^2, is reported for each regression. Two sets of response factors are given: one for a sample batch run in 2013 and another in 2014. Samples MS1, MS3, MS4, and MS5 use the response factors for 2014, while all other samples use those for 2013.

Potentially underrepresented IPLs.

Lipids with an unusual 1,2-alkanediol headgroup-backbone linkage (*e.g.* Structure **8** in Figure 1) are produced by various thermophilic bacteria including *Roseiflexus castenholzii* (van der Meer et al., 2002), and some members of *Thermomicrobia* (Pond et al., 1986; Lagutin et al., 2015), *Thermus* (Wait et al., 1997), and *Meiothermus* (Yang et al., 2006), and have been reported in lipid extracts from hot spring biomass (Zeng et al., 1992; van der Meer et al., 2002; Pancost et al., 2005, 2006). We searched for evidence of 1,2-alkanediol lipids in our samples. However, unsaturated DEGs and 1,2-alkanediols cannot be distinguished based solely on their exact masses (*e.g.* C33:1 1G-DEG and C32:0 1G-1,2-alkanediol share the same net formula of $C_{43}H_{84}O_8$ with an exact mass of 728.616 Da), and there is a possibility that these two types of lipids co-eluted during LC leading to overlapping mass spectra. As a result, we were unable to confirm the presence of 1,2-alkanediols in any sample, and the abundances of these lipids would have been grouped with unsaturated DEGs. The fraction of DEG lipids with at least one unsaturation reached between 29-51% in BP4, MS3, EP2, and OF2. If some (or all) of this actually belongs to 1,2-alkanediols, we would expect some loss of accuracy in the weighted values we calculate for nC, nUnsat, x_{ester}, x_{ether}, x_{C-C}, and Z_C for backbones and alkyl chains. However, this would not affect Z_C of IPLs because net chemical formulae and charge are shared between analogous unsaturated DEGs and 1,2-alkanediols.

Few lipids with alkyl chain hydroxylations were observed in this study. However, lipo(oligo/poly)saccharides (LOS/LPS), also known as endotoxins, are lipids rich in fatty acid chains with secondary hydroxylations in the 3' carbon position. LPS lipids are commonly produced by gram-negative bacteria and may compose up to 75% of their outer membrane (Silipo et al., 2010). LOS have also been traced to certain thermophilic bacteria (Di Lorenzo et al., 2014). These IPLs have masses exceeding 2000 Da, and as such, could not be captured by the mass spectrometry method employed in this study.

Mass spectral interpretation of novel structures.

Mass spectra and putative chemical structures of lipids identified in this work are shown in Figures S1 through S8. All lipid structures shown here are tentatively assigned based on MS/MS fragmentation patterns. Carbon positions of alkyl chain modifications, bonding, and configuration between glycolipid headgroup moieties, *etc.*, are guesses. Dotted arrows refer to inferred fragmentation of the displayed

structure and point in the direction of the fragment with the monoisotopic mass, in Da, listed at the end of the arrow. pNLC stands for 'precursor neutral loss chromatogram'. This a chromatogram based on the difference between the mass of a lipid precursor ion before and after the loss of a diagnostic fragment. Usually, in the case of IPLs, this is the headgroup or a piece of the headgroup. M+H and M-H refer to the monoisotopic mass of the displayed structure plus or minus a hydrogen atom, respectively, and assumes the resulting fragment has an overall charge of +1.

Abundance-weighted average properties of IPLs.

Table S2 gives abundance-weighted average alkyl chain properties that were calculated using Equation 2 and used in Figures 5 and 6. Table S3 reports abundance-weighted average chemical formulae of IPLs, headgroups, backbones, and alkyl chains for hot spring samples calculated using Equation 2. These average formulae were used in Equation 3 to calculate the average Z_C values depicted in Figure 7 and reported in Table S4.

Example workflow for calculating Z_C in a hypothetical sample

Here we walk through the process of calculating average properties and Z_C values for IPLs, headgroups, backbones, and alkyl chains in a hypothetical sample containing the three IPLs depicted in Figure S9. These IPLs include C80:0 1G-GDGT with no rings, C40:1 APT-DEG, and C38:2 APT-AEG.

First, mole fractions of the three IPLs are calculated using Equation 1. This requires a peak area, monoisotopic mass, and response factor value for each IPL. Peak areas and monoisotopic masses of IPL parent ions are given in Figure S9. Response factor assignments for 1G-GDGT, APT-DEG, and APT-AEG are supplied in Table 1, and values for these response factors are reported in Table S1. Assuming this hypothetical sample was analyzed in the same batch as Bison Pool, Empress Pool, and Octopus Spring (batch 1), 1G-GDGT is assigned a response factor value of 6.72×10^4, and APT-DEG and AEG are both assigned a value of 7.31×10^5. With these parameters, Equation 1 gives mole fractions of 0.661, 0.201, and 0.138 for these three IPLs, respectively.

Next, Equation 2 is used to calculate weighted sample-averaged properties such as nC, nUnsat, x_{ether}, x_{ester}, x_{GDGT}, and abundance of each element in IPLs, headgroups, backbones, and alkyl chains (carbon, hydrogen, nitrogen, oxygen, and phosphorus). This calculation requires IPL mole fractions and the total number of components and properties of interest contained within each IPL. In this case, there are eighty aliphatic carbons among four half-chains in C80 1G-GDGT, forty carbons among two chains in C40 APT-DEG, and thirty-eight carbons among two chains in C38 APT-AEG. Entering these values into Equation 2, along with the mole fractions of each IPL, results in an nC value of 19.9 aliphatic carbons per alkyl chain. One can arrive at an nUnsat value of 0.144 unsaturations per alkyl chain in the same way. Fractions of ether-linked alkyl chains, x_{ether} = 0.958, ester-linked chains, x_{ester} = 0.042, and GDGT half-chains, x_{GDGT} = 0.796, can also be derived assuming there are four ether-linked GDGT half-chains in 1G-GDGT, two ether-linked chains in APT-DEG, and one of each ether- and ester-linked chains in APT-AEG.

Average chemical formulae of IPLs and their components are also calculated with Equation 2. For instance, six carbons among two headgroups in 1G-GDGT, and five among the single headgroups of APT-DEG and AEG, gives a weighted average of 3.41 carbons per headgroup in this hypothetical sample. This value may seem low, but considering that the greatest fraction belongs to 1G-GDGT, and that this IPL has six carbons split between two headgroups, it follows that the weighted average is close to three carbons per headgroup. One obtains an average headgroup formula of $C_{3.41}H_{7.43}N_{0.204}O_{3.21}P_{0.204}$ after repeating this process for each element. Likewise, the average chemical formula for backbones is $C_3H_5O_3$,

and $C_{19.9}H_{39.7}O_{0.0416}$ for alkyl chains. The average chemical formula for IPLs in this hypothetical sample is $C_{76.8}H_{152.0}N_{0.339}O_{10.7}P_{0.339}$ by assuming the number of components in a full IPL is equal to one in Equation 2. Lastly, average chemical formulae are entered into Equation 3 to obtain Z_C values of -1.71 for IPLs, -0.413 for headgroups, 0.333 for backbones, and -1.99 for alkyl chains.

REFERENCES

Di Lorenzo, F., Paciello, I., Fazio, L. L., Albuquerque, L., Sturiale, L., da Costa, M. S., et al. (2014). Thermophiles as potential source of novel endotoxin antagonists: the full structure and bioactivity of the lipo-oligosaccharide from *Thermomonas hydrothermalis*. *ChemBioChem* 15, 2146–2155

Lagutin, K., MacKenzie, A., Houghton, K. M., Stott, M. B., and Vyssotski, M. (2015). Novel long-chain diol phospholipids from some bacteria belonging to the class *Thermomicrobia*. *Lipids* 50, 303–311

Pancost, R. D., Pressley, S., Coleman, J. M., Benning, L. G., and Mountain, B. W. (2005). Lipid biomolecules in silica sinters: indicators of microbial biodiversity. *Environmental Microbiology* 7, 66–77

Pancost, R. D., Pressley, S., Coleman, J. M., Talbot, H. M., Kelly, S. P., Farrimond, P., et al. (2006). Composition and implications of diverse lipids in New Zealand geothermal sinters. *Geobiology* 4, 71–92

Pond, J. L., Langworthy, T. A., and Holzer, G. (1986). Long-chain diols: a new class of membrane lipids from a thermophilic bacterium. *Science* 231, 1134–1136

Silipo, A., De Castro, C., Lanzetta, R., Parrilli, M., and Molinaro, A. (2010). Lipopolysaccharides. In *Prokaryotic Cell Wall Compounds: Structure and Biochemistry*, eds. H. König, H. Claus, and A. Varma (Berlin, Heidelberg: Springer). 133–153

van der Meer, M. T., Schouten, S., Hanada, S., Hopmans, E. C., Damsté, J. S., and Ward, D. M. (2002). Alkane-1, 2-diol-based glycosides and fatty glycosides and wax esters in *Roseiflexus castenholzii* and hot spring microbial mats. *Archives of Microbiology* 178, 229–237

Wait, R., Carreto, L., Nobre, M. F., Ferreira, A. M., and da Costa, M. S. (1997). Characterization of novel long-chain 1, 2-diols in *Thermus* species and demonstration that *Thermus* strains contain both glycerol-linked and diol-linked glycolipids. *Journal of Bacteriology* 179, 6154–6162

Yang, Y.-L., Yang, F.-L., Jao, S.-C., Chen, M.-Y., Tsay, S.-S., Zou, W., et al. (2006). Structural elucidation of phosphoglycolipids from strains of the bacterial thermophiles *Thermus* and *Meiothermus*. *Journal of Lipid Research* 47, 1823–1832

Zeng, Y. B., Ward, D. M., Brassell, S. C., and Eglinton, G. (1992). Biogeochemistry of hot spring environments: 2. Lipid compositions of Yellowstone (Wyoming, USA) cyanobacterial and *Chloroflexus* mats. *Chemical Geology* 95, 327–345

2 SUPPLEMENTARY TABLES AND FIGURES

Table S1. HPLC-MS IPL standards and response factors.

					Batch 1[b]		Batch 2[c]	
No.[a]	Standard	Chains	Full product name	Supplier	RF	R^2	RF	R^2
1	1Gly-DAG	mix of C34:6, C36:6	1,2-diacyl-3-O-(a-D-glucopyranosyl)-sn-glycerol (E.coli)	Avanti Polar Lipids, USA	$6.37\cdot10^5$	0.95	$9.45\cdot10^5$	0.99
2	1Gly-GDGT-PG	mix of 0-3 rings, ∼20% H-shaped	Main phospholipid of *Thermoplasma acidophilum* (>95% pure)	Matreya LLC, USA	$6.72\cdot10^4$	0.96	$1.16\cdot10^4$	0.91
3	PC-DAG	C42:0	1,2-diheneicosanoyl-sn-glycero-3-phosphocholine	Avanti Polar Lipids, USA	$7.31\cdot10^5$	0.99	$2.71\cdot10^5$	0.91
4	2Gly-DAG	mix of C34:2, C34:3 C34:6, C36:6	Digalactosyldiacylglcyerol (plant, hydrogenated)	Avanti Polar Lipids, USA	$1.33\cdot10^5$	0.99	$9.90\cdot10^4$	0.99
5	SQ-DAG	mix of C34:2, C34:3, C36:6	Sulfoquinovosyldiacylglycerol	Avanti Polar Lipids, USA	$1.31\cdot10^5$	0.95	$1.90\cdot10^5$	0.99
6	PI-DAG	C32:0	1,2-dipalmitoyl-sn-glycero-3-phospho-(1'-myo-inositol) (ammonium salt)	Avanti Polar Lipids, USA	$7.94\cdot10^3$	0.88	$5.95\cdot10^3$	0.99
7	DPG	C72:4	1',3'-bis[1,2-dioleoyl-sn-glycero-3-phospho]-glycerol (sodium salt)	Avanti Polar Lipids, USA	$3.97\cdot10^5$	0.95	$7.24\cdot10^4$	0.99
8	PDME-DAG	C32:0	1,2-dipalmitoyl-sn-glycero-3-phosphoethanolamine-N,N-dimethyl	Avanti Polar Lipids, USA	$1.11\cdot10^6$	0.97	$1.96\cdot10^6$	0.99
9	PE-DAG	C32:0	1,2-dipalmitoyl-sn-glycero-3-phosphoethanolamine	Avanti Polar Lipids, USA	$4.06\cdot10^5$	0.99	$2.92\cdot10^5$	0.95
10	PG-DAG	C32:0	1,2-dipalmitoyl-sn-glycero-3-phospho-(1'-rac-glycerol)	Avanti Polar Lipids, USA	$1.49\cdot10^5$	0.98	$1.02\cdot10^5$	0.99
11	PME-DAG	C32:0	1,2-dipalmitoyl-sn-glycero-3-phosphoethanolamine-N-methyl	Avanti Polar Lipids, USA	$5.45\cdot10^5$	0.94	$5.93\cdot10^5$	0.99
12	PS-DAG	C32:0	1,2-dipalmitoyl-sn-glycero-3-phospho-L-serine (sodium salt)	Avanti Polar Lipids, USA	$2.03\cdot10^4$	0.98	$2.30\cdot10^4$	0.97
13	DGTS-d9	C32:0	1,2-dipalmitoyl-sn-glycero-3-O-4'-[N,N,N-trimethyl(d9)]-homoserine	Avanti Polar Lipids, USA	$1.65\cdot10^6$	0.89	$7.16\cdot10^6$	0.96

[a] Corresponds to numbered standards in Table 1 of the main text.

[b] Batch 1 was analyzed in 2013 and includes all Bison Pool, Empress Pool, and Octopus Spring samples, as well as Mound Spring sample MS2. Linear response factors and R^2 taken from two replicate calibrations of 0.1, 0.5, 1, 5, 10, and 50 ng standard injections.

[c] Batch 2 was analyzed in 2014 and includes Mound Spring samples MS1, MS3, MS4, and MS5. Linear response factors and R^2 taken from a single calibration of 0.1, 0.5, 1, 5, and 10 ng standard injections.

Table S2. Summary of abundance-weighted average alkyl chain properties.

Site	Sample	nC	nUnsat	nOH	Mole fraction linkage type					Rings per
					x_{ether}	x_{ester}	x_{amide}	x_{C-C}	x_{GDGT}	GDGT
Bison	BP1	19.80	$3.12\cdot10^{-1}$	$2.10\cdot10^{-3}$	$7.03\cdot10^{-1}$	$2.75\cdot10^{-1}$	$1.11\cdot10^{-2}$	$1.09\cdot10^{-2}$	$4.90\cdot10^{-1}$	1.8
Pool	BP2	19.39	$3.62\cdot10^{-1}$	$5.27\cdot10^{-3}$	$5.46\cdot10^{-1}$	$4.18\cdot10^{-1}$	$1.64\cdot10^{-2}$	$1.92\cdot10^{-2}$	$3.00\cdot10^{-1}$	2.2
	BP3	17.94	$3.34\cdot10^{-1}$	$5.91\cdot10^{-3}$	$2.17\cdot10^{-1}$	$7.32\cdot10^{-1}$	$2.37\cdot10^{-2}$	$2.79\cdot10^{-2}$	$8.81\cdot10^{-2}$	2.8
	BP4	17.28	$3.46\cdot10^{-1}$	$4.70\cdot10^{-3}$	$4.14\cdot10^{-1}$	$5.52\cdot10^{-1}$	$1.51\cdot10^{-2}$	$1.88\cdot10^{-2}$	$7.16\cdot10^{-3}$	3.1
	BP5	16.82	$4.91\cdot10^{-1}$	$2.93\cdot10^{-4}$	$9.80\cdot10^{-2}$	$8.46\cdot10^{-1}$	$2.83\cdot10^{-3}$	$5.33\cdot10^{-2}$	$7.20\cdot10^{-4}$	3.2
	BP6	16.68	$8.10\cdot10^{-1}$	$3.03\cdot10^{-3}$	$3.28\cdot10^{-3}$	$9.72\cdot10^{-1}$	$3.36\cdot10^{-3}$	$2.15\cdot10^{-2}$	$2.94\cdot10^{-3}$	3.3
Mound	MS1	20.00	$2.72\cdot10^{-4}$	-	< 1.00	$3.04\cdot10^{-4}$	$4.78\cdot10^{-5}$	$4.96\cdot10^{-5}$	$9.99\cdot10^{-1}$	2.5
Spring	MS2	19.60	$1.94\cdot10^{-1}$	$7.79\cdot10^{-4}$	$7.39\cdot10^{-1}$	$1.71\cdot10^{-1}$	$4.74\cdot10^{-2}$	$4.33\cdot10^{-2}$	$6.07\cdot10^{-1}$	2.0
	MS3	18.01	$3.53\cdot10^{-1}$	$7.06\cdot10^{-4}$	$6.54\cdot10^{-1}$	$3.15\cdot10^{-1}$	$1.55\cdot10^{-2}$	$1.50\cdot10^{-2}$	$1.64\cdot10^{-1}$	2.8
	MS4	17.38	$3.88\cdot10^{-1}$	$1.48\cdot10^{-4}$	$1.94\cdot10^{-1}$	$7.84\cdot10^{-1}$	$5.10\cdot10^{-3}$	$1.69\cdot10^{-2}$	$5.85\cdot10^{-2}$	2.9
	MS5	16.78	$9.65\cdot10^{-1}$	$3.39\cdot10^{-5}$	$2.68\cdot10^{-2}$	$9.47\cdot10^{-1}$	$3.52\cdot10^{-3}$	$2.23\cdot10^{-2}$	$1.09\cdot10^{-2}$	3.5
Empress	EP1	19.83	$1.51\cdot10^{-2}$	$2.35\cdot10^{-4}$	$9.83\cdot10^{-1}$	$1.47\cdot10^{-2}$	$1.11\cdot10^{-3}$	$1.13\cdot10^{-3}$	$8.63\cdot10^{-1}$	2.1
Pool	EP2	18.91	$2.24\cdot10^{-1}$	$1.34\cdot10^{-2}$	$7.06\cdot10^{-1}$	$2.30\cdot10^{-1}$	$3.15\cdot10^{-2}$	$3.25\cdot10^{-2}$	$4.71\cdot10^{-1}$	2.5
	EP3	19.06	$1.37\cdot10^{-1}$	$6.14\cdot10^{-3}$	$6.88\cdot10^{-1}$	$2.85\cdot10^{-1}$	$1.07\cdot10^{-2}$	$1.65\cdot10^{-2}$	$6.06\cdot10^{-1}$	2.5
	EP4	17.78	$3.97\cdot10^{-1}$	$3.88\cdot10^{-3}$	$2.84\cdot10^{-1}$	$6.72\cdot10^{-1}$	$5.91\cdot10^{-3}$	$3.75\cdot10^{-2}$	$2.31\cdot10^{-1}$	2.5
	EP5	17.08	$6.87\cdot10^{-1}$	$4.47\cdot10^{-4}$	$1.32\cdot10^{-1}$	$8.11\cdot10^{-1}$	$7.42\cdot10^{-4}$	$5.62\cdot10^{-2}$	$1.26\cdot10^{-1}$	2.3
Octopus	OS1	20.42	$1.86\cdot10^{-1}$	$2.50\cdot10^{-4}$	$7.38\cdot10^{-1}$	$2.55\cdot10^{-1}$	$3.24\cdot10^{-3}$	$3.28\cdot10^{-3}$	$4.21\cdot10^{-1}$	1.1
Spring	OS2	17.52	$3.17\cdot10^{-1}$	$5.41\cdot10^{-3}$	$4.10\cdot10^{-1}$	$5.64\cdot10^{-1}$	$8.71\cdot10^{-3}$	$1.73\cdot10^{-2}$	$7.60\cdot10^{-3}$	2.3

Table S3. Abundance-weighted average chemical formulae of IPLs and their component parts.

IPLs	Headgroups	Backbones	Alkyl chains
$C_{64.1}H_{123.}N_{2.15e-1}O_{12.3}P_{5.01e-1}S_{1.27e-3}^{+8.04e-3}$	$C_{5.61}H_{11.0}N_{1.41e-1}O_{5.70}P_{3.95e-1}S_{9.55e-4}^{+6.07e-3}$	$C_{3.02}H_{5.06}N_{2.19e-2}O_{2.98}$	$C_{19.8}H_{38.5}O_{2.85e-1}$
$C_{56.9}H_{109.}N_{2.10e-1}O_{12.3}P_{6.07e-1}S_{2.42e-3}^{+1.42e-2}$	$C_{6.25}H_{12.1}N_{1.42e-1}O_{6.63}P_{5.34e-1}S_{2.05e-3}^{+1.21e-2}$	$C_{3.04}H_{5.08}N_{3.86e-2}O_{2.97}$	$C_{19.4}H_{37.6}O_{4.36e-1}$
$C_{48.0}H_{91.5}N_{1.48e-1}O_{12.1}P_{5.31e-1}S_{8.32e-2}^{+4.61e-3}$	$C_{6.71}H_{12.9}N_{8.54e-2}O_{7.12}P_{5.12e-1}S_{7.95e-2}^{+4.40e-3}$	$C_{3.06}H_{5.13}N_{5.61e-2}O_{2.96}$	$C_{17.9}H_{34.5}O_{7.54e-1}$
$C_{45.1}H_{86.8}N_{8.39e-2}O_{12.1}P_{6.25e-1}S_{5.38e-2}^{+8.07e-3}$	$C_{7.22}H_{13.7}N_{4.59e-2}O_{7.91}P_{6.23e-1}S_{5.36e-2}^{+8.04e-3}$	$C_{3.04}H_{5.10}N_{3.76e-2}O_{2.97}$	$C_{17.3}H_{33.7}O_{5.67e-1}$
$C_{44.6}H_{83.7}N_{3.32e-1}O_{11.4}P_{2.17e-1}S_{1.21e-1}^{+1.24e-1}$	$C_{7.82}H_{14.5}N_{2.25e-1}O_{6.79}P_{2.17e-1}S_{1.21e-1}^{+1.24e-1}$	$C_{3.11}H_{5.12}N_{1.07e-1}O_{2.91}$	$C_{16.8}H_{32.0}O_{8.49e-1}$
$C_{43.5}H_{80.4}N_{2.70e-1}O_{11.6}P_{4.64e-1}S_{1.18e-1}^{+8.39e-2}$	$C_{6.67}H_{13.0}N_{2.26e-1}O_{6.60}P_{4.63e-1}S_{1.18e-1}^{+8.37e-2}$	$C_{3.04}H_{5.06}N_{4.30e-2}O_{2.96}$	$C_{16.7}H_{30.8}O_{9.75e-1}$
$C_{92.2}H_{177.}N_{4.49e-4}O_{11.2}P_{2.43e-4}^{+7.34e-6}$	$C_{3.14}H_{6.23}N_{1.25e-4}O_{2.61}P_{1.22e-4}^{+3.67e-6}$	$C_{3.00}H_{5.00}N_{9.93e-5}O_{3.00}$	$C_{20.0}H_{38.8}O_{3.52e-4}$
$C_{68.7}H_{133.}N_{2.22e-1}O_{12.0}P_{3.77e-1}S_{2.10e-2}^{+4.40e-3}$	$C_{5.12}H_{9.86}N_{6.80e-2}O_{4.99}P_{2.65e-1}S_{1.46e-2}^{+3.06e-3}$	$C_{3.09}H_{5.25}N_{8.73e-2}O_{2.92}$	$C_{19.6}H_{38.2}O_{2.12e-1}$
$C_{49.9}H_{96.5}N_{4.98e-2}O_{11.9}P_{6.47e-1}S_{2.03e-2}^{+1.27e-3}$	$C_{6.57}H_{12.6}N_{1.57e-2}O_{7.26}P_{5.94e-1}S_{1.86e-2}^{+1.17e-3}$	$C_{3.03}H_{5.09}N_{3.00e-2}O_{2.97}$	$C_{18.0}H_{35.3}O_{3.30e-1}$
$C_{47.2}H_{89.6}N_{2.07e-1}O_{12.1}P_{4.93e-1}S_{5.34e-2}^{+5.00e-2}$	$C_{7.20}H_{13.8}N_{1.67e-1}O_{7.13}P_{4.78e-1}S_{5.18e-2}^{+4.85e-2}$	$C_{3.03}H_{5.05}N_{3.38e-2}O_{2.97}$	$C_{17.4}H_{33.3}O_{7.89e-1}$
$C_{46.6}H_{85.5}N_{2.60e-1}O_{12.5}P_{3.65e-1}S_{1.25e-1}^{+1.69e-1}$	$C_{8.29}H_{15.9}N_{2.12e-1}O_{7.36}P_{3.62e-1}S_{1.24e-1}^{+1.68e-1}$	$C_{3.04}H_{5.06}N_{4.46e-2}O_{2.96}$	$C_{16.8}H_{30.7}O_{9.51e-1}$
$C_{85.0}H_{164.}N_{1.17e-1}O_{13.8}P_{4.38e-2}^{+2.05e-4}$	$C_{5.66}H_{10.4}N_{6.51e-2}O_{4.79}P_{2.97e-2}^{+1.16e-4}$	$C_{3.00}H_{5.01}N_{2.27e-3}O_{3.00}$	$C_{19.8}H_{38.8}O_{1.61e-2}$
$C_{62.0}H_{119.}N_{1.87e-1}O_{12.5}P_{4.18e-1}S_{1.16e-2}^{+8.26e-3}$	$C_{6.22}H_{11.8}N_{7.79e-2}O_{6.07}P_{3.28e-1}S_{8.81e-3}^{+6.30e-3}$	$C_{3.07}H_{5.18}N_{6.52e-2}O_{2.94}$	$C_{18.9}H_{36.8}O_{2.61e-1}$
$C_{67.9}H_{130.}N_{1.06e-1}O_{13.4}P_{3.20e-1}S_{3.36e-2}^{+1.15e-2}$	$C_{6.11}H_{11.5}N_{4.11e-2}O_{5.75}P_{2.32e-1}S_{2.34e-2}^{+7.98e-3}$	$C_{3.03}H_{5.07}N_{3.31e-2}O_{2.97}$	$C_{19.1}H_{36.9}O_{2.95e-1}$
$C_{51.6}H_{97.2}N_{2.52e-1}O_{11.7}P_{1.99e-1}S_{1.13e-1}^{+8.90e-2}$	$C_{6.88}H_{12.8}N_{1.48e-1}O_{6.06}P_{1.78e-1}S_{9.96e-2}^{+7.87e-2}$	$C_{3.07}H_{5.10}N_{7.50e-2}O_{2.93}$	$C_{17.8}H_{33.9}O_{6.78e-1}$
$C_{48.1}H_{89.0}N_{3.69e-1}O_{11.6}P_{8.99e-2}S_{1.79e-1}^{+1.57e-1}$	$C_{7.78}H_{14.4}N_{2.33e-1}O_{6.34}P_{8.53e-2}S_{1.68e-1}^{+1.47e-1}$	$C_{3.11}H_{5.12}N_{1.12e-1}O_{2.90}$	$C_{17.1}H_{31.9}O_{8.12e-1}$
$C_{64.4}H_{125.}N_{9.12e-2}O_{13.7}P_{5.91e-1}S_{2.83e-3}^{+1.45e-3}$	$C_{6.97}H_{13.3}N_{8.08e-2}O_{7.32}P_{5.36e-1}S_{2.23e-3}^{+1.15e-3}$	$C_{3.01}H_{5.02}N_{6.57e-3}O_{2.99}$	$C_{20.4}H_{40.3}O_{2.63e-1}$
$C_{45.2}H_{87.3}N_{1.01e-1}O_{11.9}P_{6.79e-1}S_{4.19e-2}^{+1.93e-2}$	$C_{6.91}H_{13.3}N_{6.61e-2}O_{7.73}P_{6.78e-1}S_{4.18e-2}^{+1.93e-2}$	$C_{3.03}H_{5.07}N_{3.47e-2}O_{2.97}$	$C_{17.5}H_{34.2}O_{5.73e-1}$

Note: fractional charge is represented by a + followed by its fraction.

Table S4. Z_C of IPLs and their component parts.

Site	Sample	IPLs	Headgroups	Backbones	Alkyl chains
Bison	BP1	-1.56	-0.20	0.32	-1.92
Pool	BP2	-1.53	-0.17	0.32	-1.89
	BP3	-1.45	-0.19	0.31	-1.84
	BP4	-1.46	-0.15	0.31	-1.88
	BP5	-1.38	-0.22	0.33	-1.80
	BP6	-1.36	-0.27	0.33	-1.73
Mound	MS1	-1.68	-0.32	0.33	-1.94
Spring	MS2	-1.61	-0.21	0.28	-1.93
	MS3	-1.52	-0.16	0.31	-1.92
	MS4	-1.43	-0.22	0.33	-1.82
	MS5	-1.33	-0.32	0.33	-1.71
Empress	EP1	-1.60	-0.14	0.33	-1.96
Pool	EP2	-1.54	-0.18	0.29	-1.92
	EP3	-1.54	-0.18	0.32	-1.90
	EP4	-1.44	-0.21	0.32	-1.83
	EP5	-1.37	-0.25	0.33	-1.77
Octopus	OS1	-1.56	-0.16	0.33	-1.95
Spring	OS2	-1.48	-0.17	0.32	-1.89

2.1 Figures

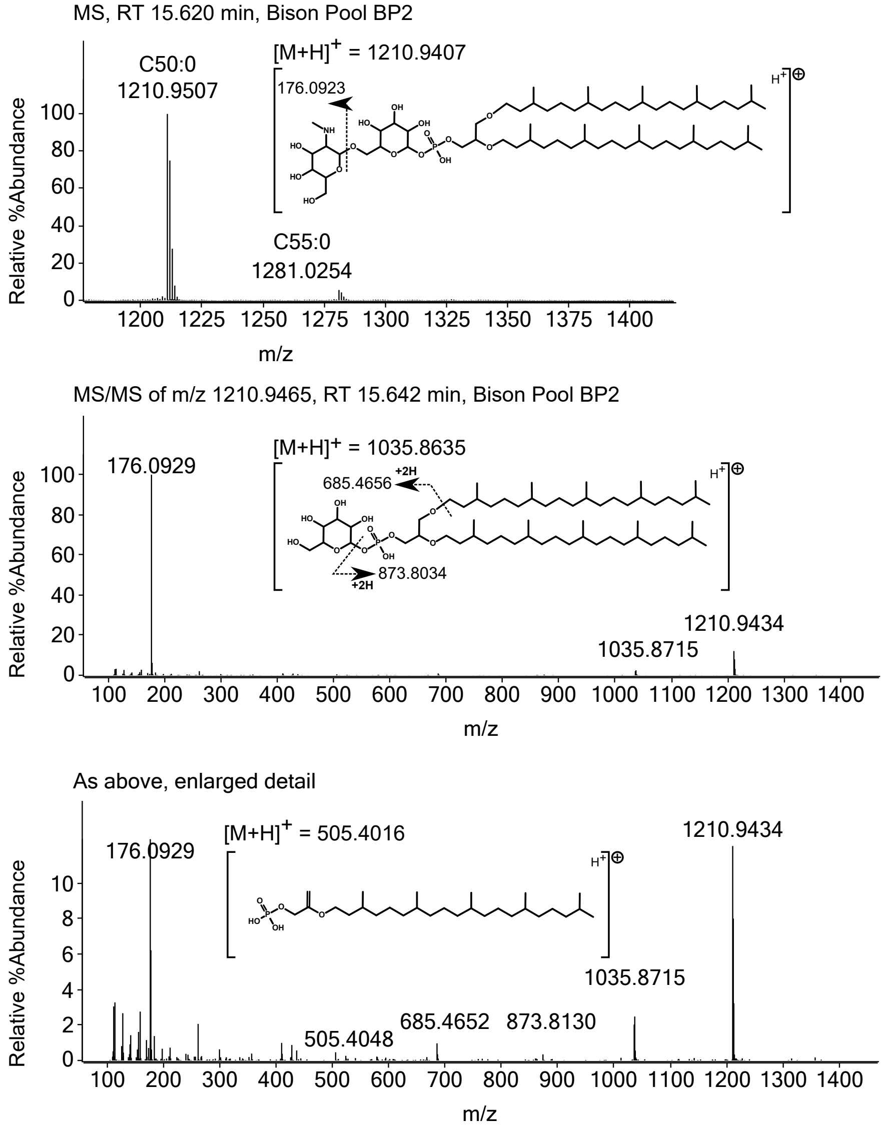

Figure S1. Mass spectra at the MS (top panel) and MS/MS (middle and bottom panels) levels and putative structure and fragments for the (N-methyl)glycosaminyl monoglycosyl phosphatidylarchaeol (MeNG-G-P-AR). The parent ion of the C50:0 variant is depicted in the top panel and resulting MS/MS spectra and possible fragment structures are shown in the middle and bottom panels.

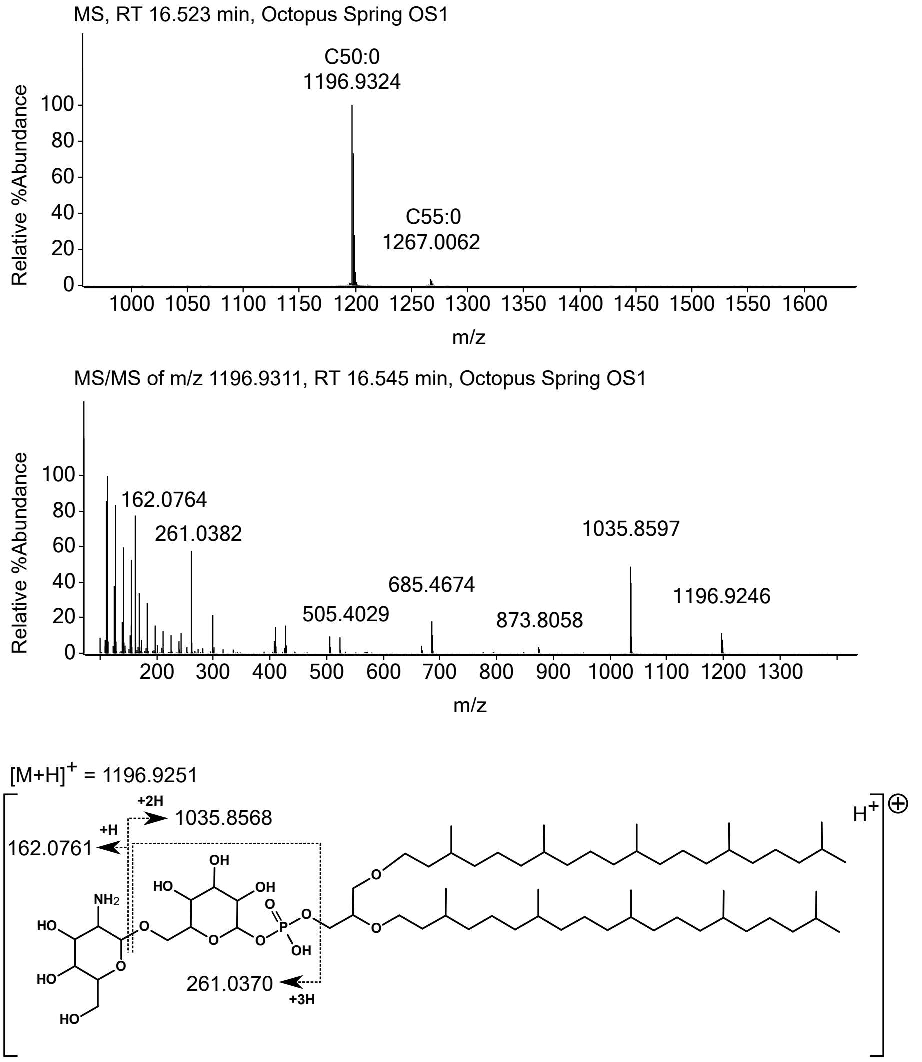

Figure S2. Mass spectra at the MS (top panel) and MS/MS (middle panel) levels and putative structure for the C:50 variant of (N)glycosaminyl monoglycosyl phosphatidylarchaeol (NG-G-P-AR). This lipid shares many MS/MS fragments with MeNG-G-P-AR (Figure S1) due to their structural similarity, differing only by a methyl group on the terminal headgroup moiety.

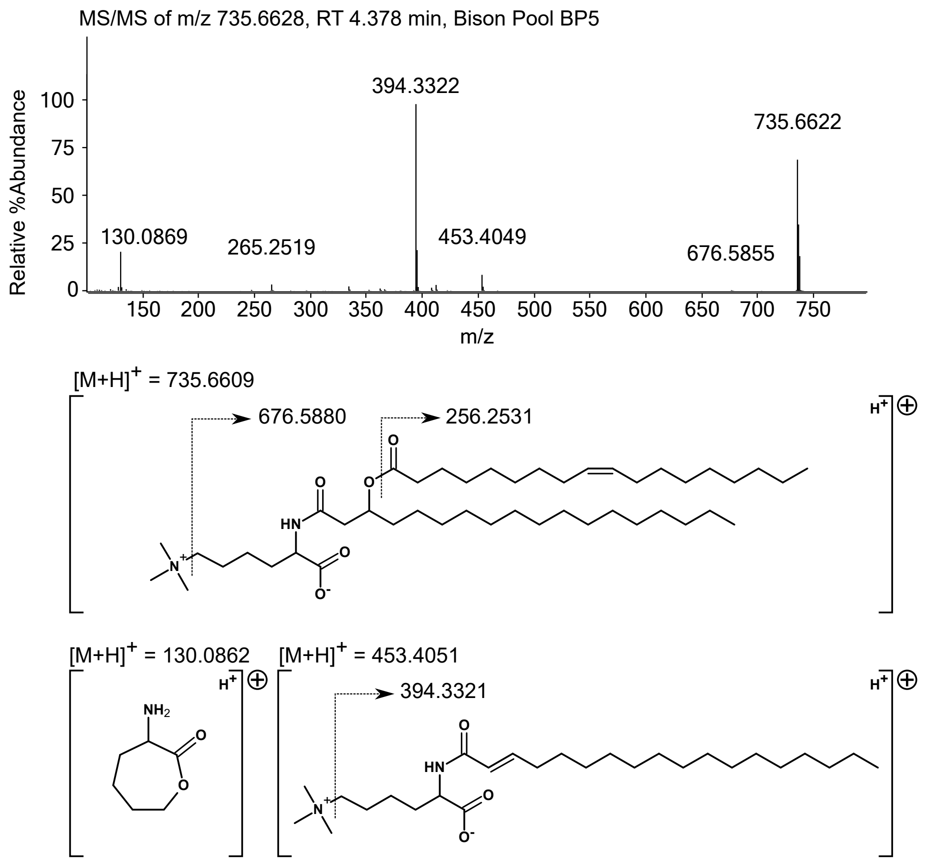

Figure S3. Mass spectrum at MS/MS level and putative structure and fragments for (6-N,6-N,6-N)trimethyllysine lipid (TM-KL). The headgroup is depicted in its zwitterionic form to indicate how its m/z was interpreted with a proton adduct. The headgroup formula for TM-KL is given in Table 1 as $C_9H_{19}NO_2^+$ for its fully protonated state, which differs from its zwitterionic formula of $C_9H_{18}NO_2$. Differences in protonation state do not affect Z_C.

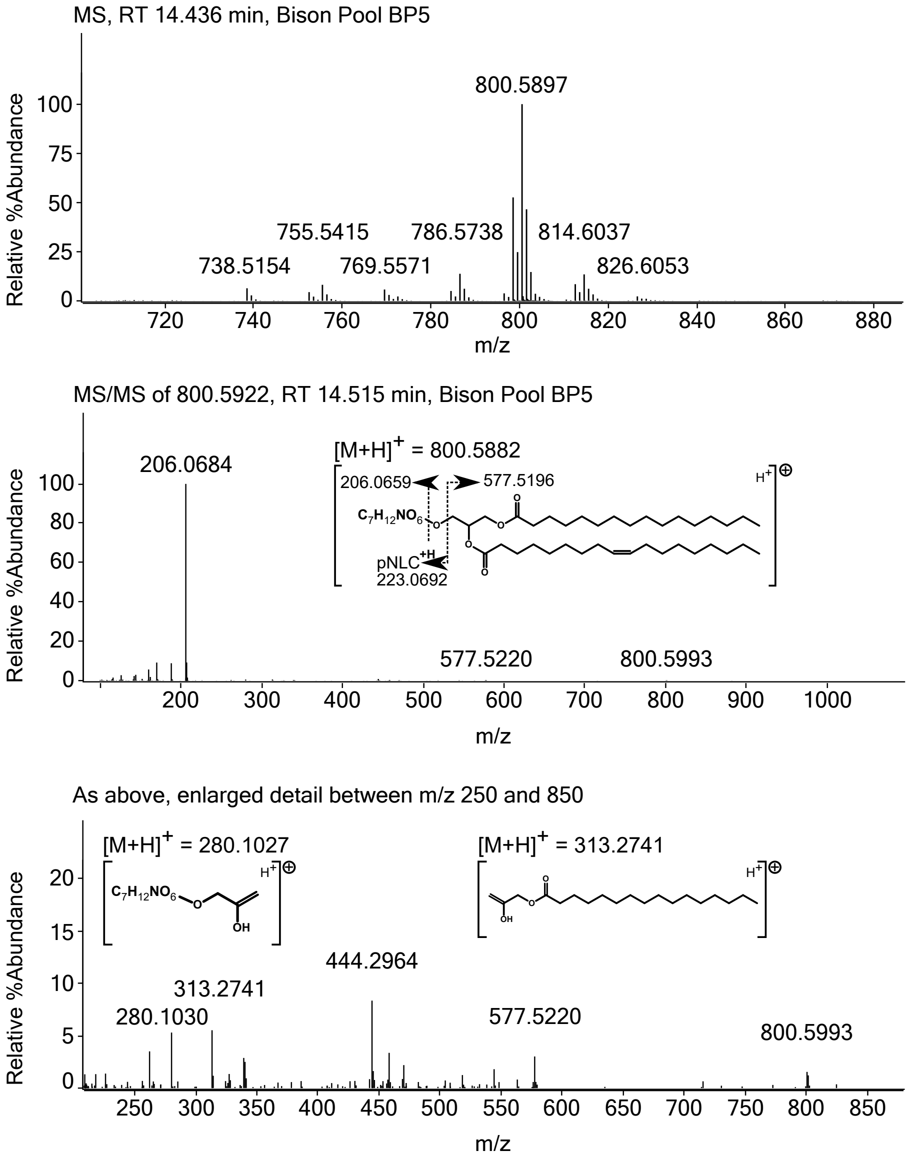

Figure S4. Mass spectra at the MS (top panel) and MS/MS (bottom and middle panels) levels for unknown lipid '223'-DAG. The headgroup is indicated by $C_7H_{12}NO_6$, our best guess for the chemical formula based on fragmentation patterns.

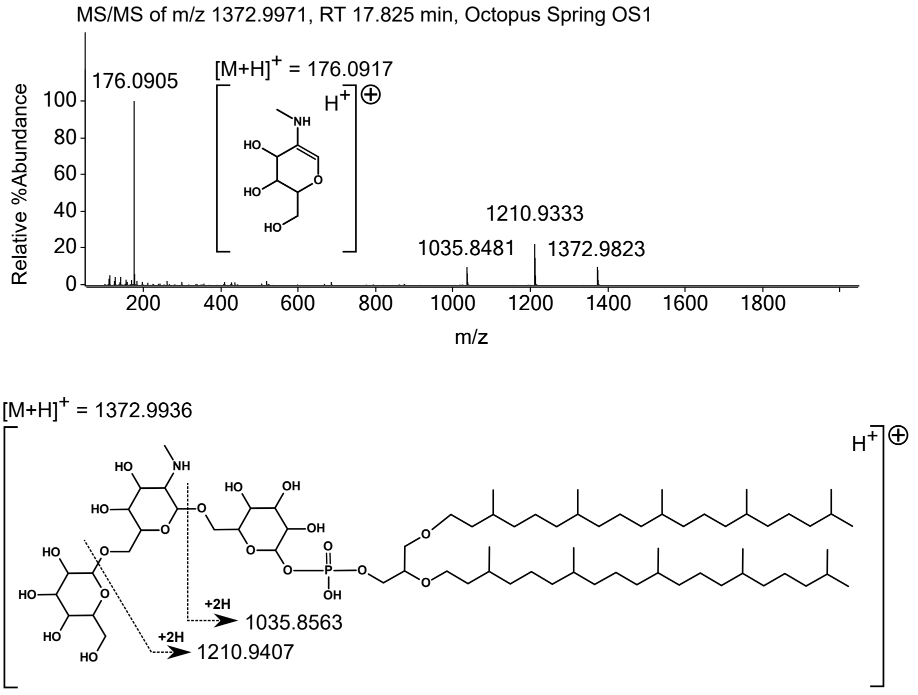

Figure S5. Mass spectrum at the MS/MS level and putative structure and fragments for glycosyl (N-methyl)glycosaminyl glycosyl phosphatidylarchaeol (G-MeNG-G-P-AR).

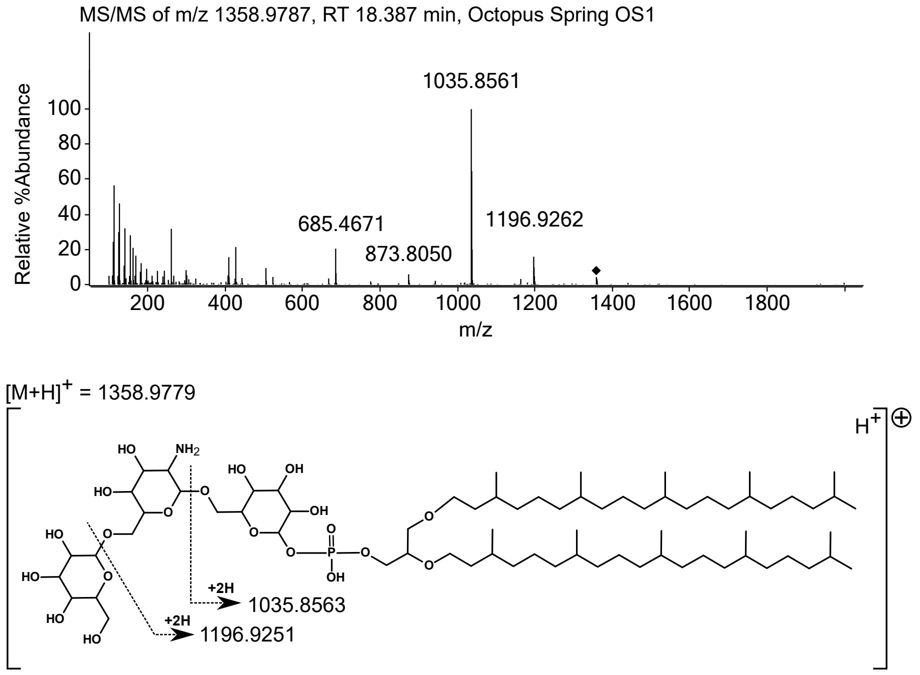

Figure S6. Mass spectrum at the MS/MS level and putative structure for glycosyl (N)glycosaminyl glycosyl phosphatidylarchaeol (G-NG-G-P-AR). See Figure S1 (middle panel) for putative fragment structures proposed for peaks m/z 1035, 685, and 873.

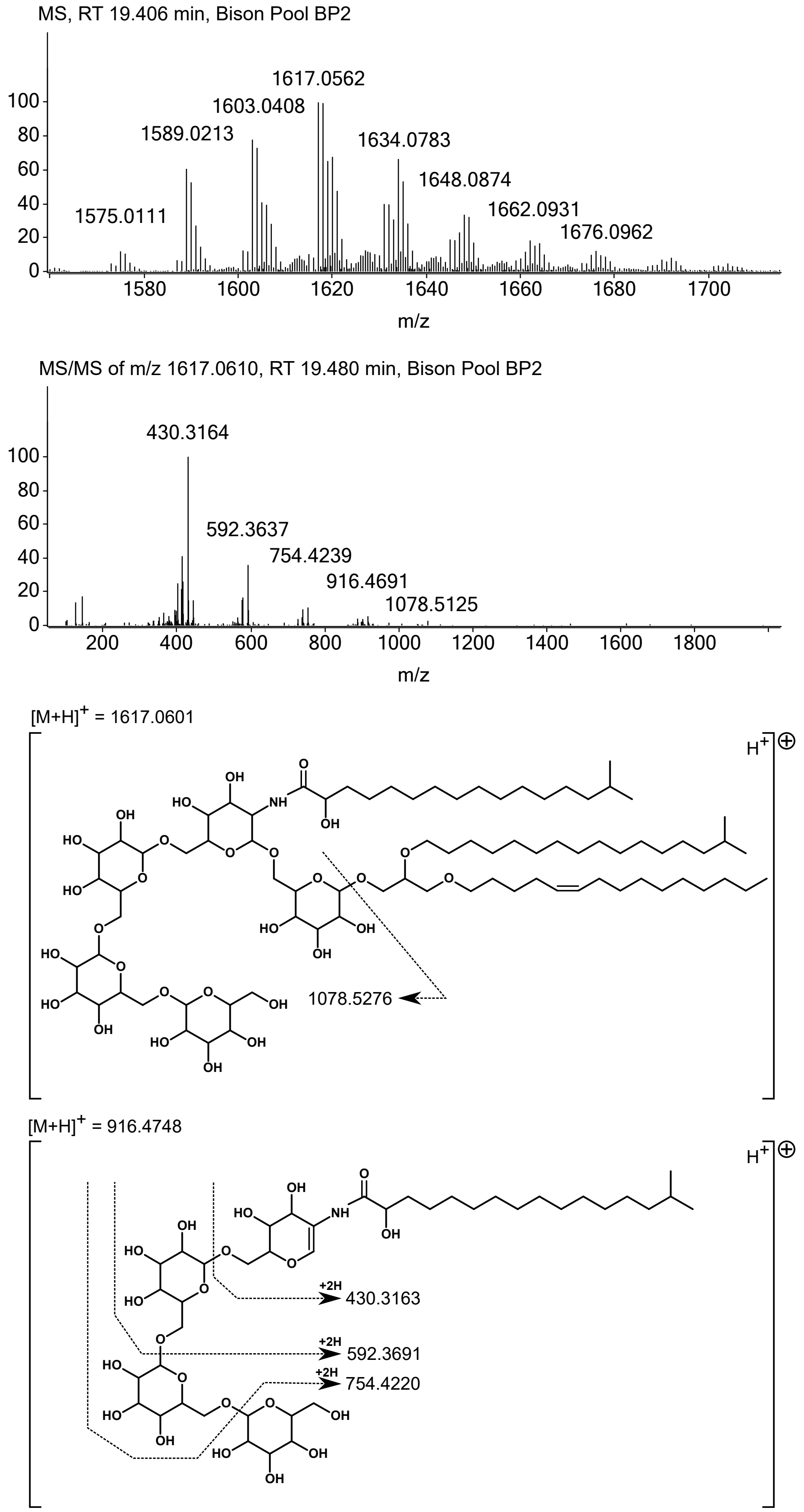

Figure S7. Mass spectra at the MS and MS/MS levels (top panels) and putative structure and fragments (bottom panels) for triglycosyl (N-acetyl)glycosaminyl glycosyl dietherglycerol (3G-NAcG-G-DEG).

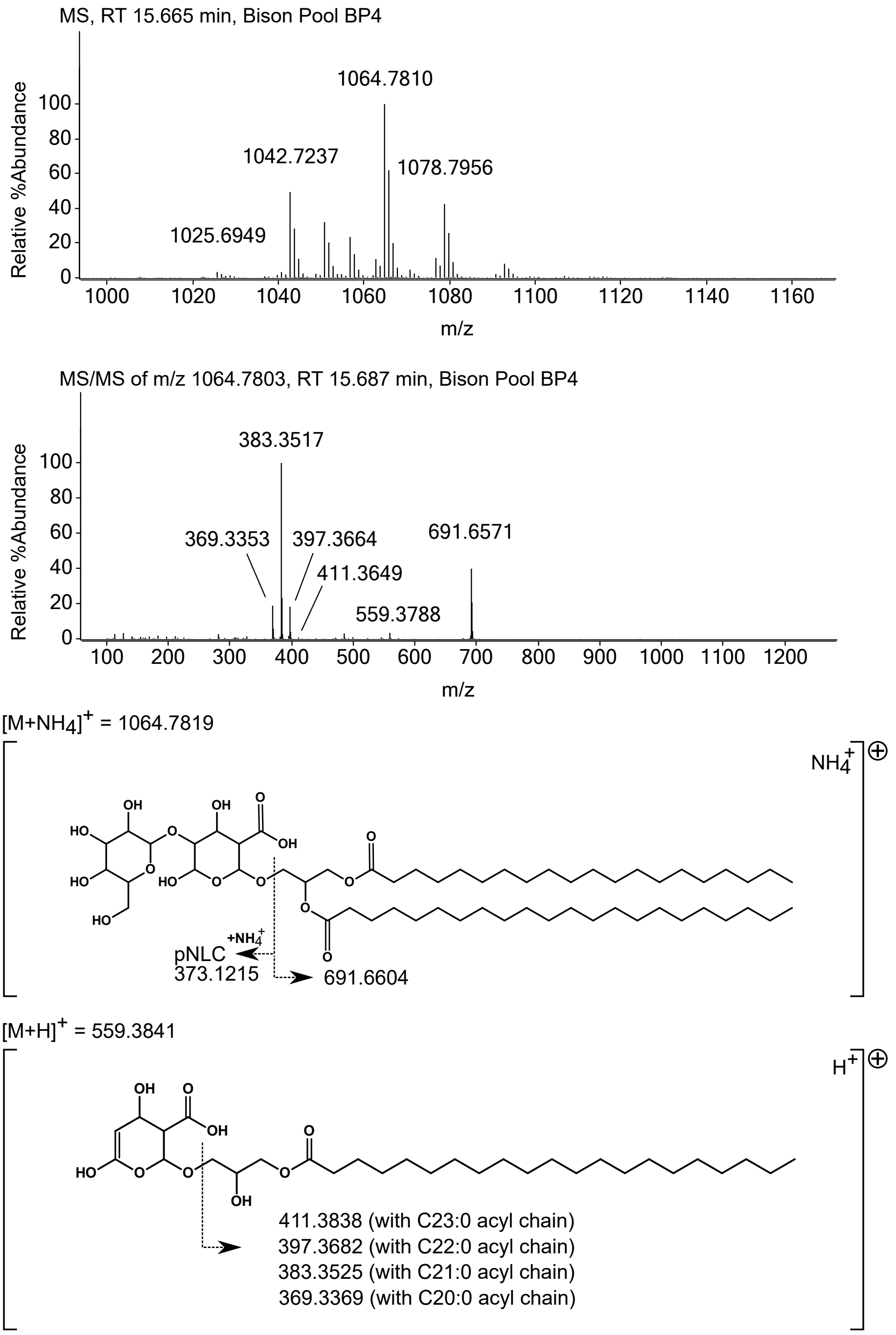

Figure S8. Mass spectra at the MS (top panel) and MS/MS (second panel) levels and putative structure and fragments (bottom panels) for G-GA-DAG.

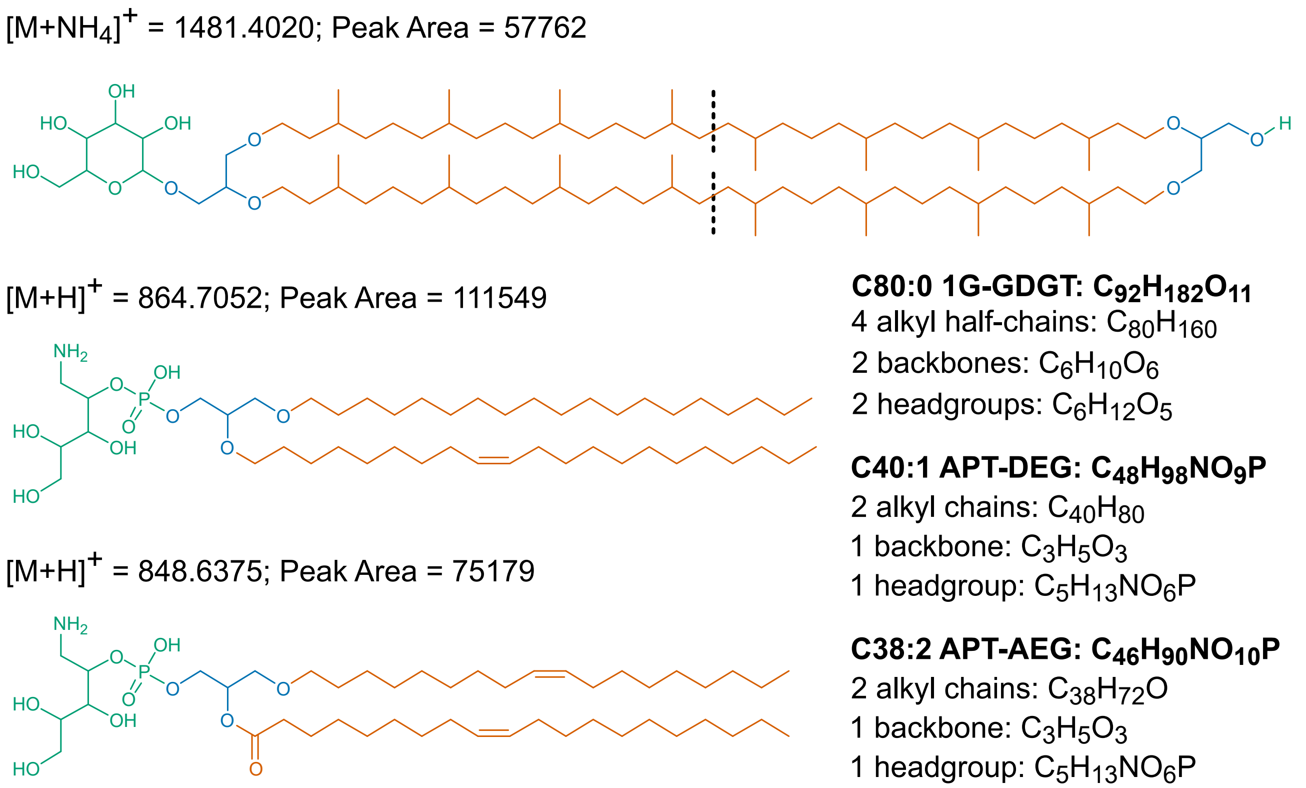

Figure S9. IPLs, structural divisions, and peak areas used in the workflow example for a hypothetical sample (see text). From top to bottom, the IPLs shown are C80:0 1G-GDGT, C40:1 APT-DEG, and C38:2 APT-AEG. Headgroups (green), backbones (blue), and alkyl chains (orange) are designated according to the IPL component division criteria used in this study. Note that the hydrogen atom on the right side of 1G-GDGT is shown as a headgroup. The dashed black lines indicate where the two membrane-spanning biphytyl chains of 1G-GDGT are divided into four alkyl half-chains during calculation of weighted chain properties, elemental abundance, and Z_C.
